# Individual and organizational interventions to reduce burnout in resident physicians: a systematic review and meta-analysis

**DOI:** 10.1186/s12909-024-06195-3

**Published:** 2024-10-30

**Authors:** Wuttipat Kiratipaisarl, Vithawat Surawattanasakul, Wachiranun Sirikul

**Affiliations:** 1 Department of Community Medicine, Faculty of Medicine, Chiang Mai University, Chiang Mai, Thailand; 2 Environmental Medicine and Occupational Medicine Excellence Center, Faculty of Medicine, Chiang Mai University, Chiang Mai, Thailand

**Keywords:** Burnout, professional, Occupational stress, Controlled clinical trial, Occupational health, Environment health, Internship, Residency

## Abstract

**Background:**

Burnout among resident physicians during training has been prevalent, prompting training centers to introduce interventions at the individual or organizational level. However, empirical evidence is crucial before implementing such programs in practice.

**Methods:**

A systematic review and meta-analysis was carried out to evaluate the effectiveness of individual and organizational interventions in reducing burnout among resident physicians. Searching was done across five databases—PubMed, Scopus, ScienceDirect, Embase, and Cochrane Library from 1 December 2023 to 26 August 2024. The Preferred Reporting Items for Systematic Reviews and Meta-Analyses (PRISMA) statement was used for our reporting of study selection process. Eligibility criteria were randomized or non-randomized designs, with prospective intervention, with a comparator group focused on individual or organizational interventions reducing burnout, in any language and publication date. The Maslach Burnout Inventory scores for emotional exhaustion (EE), depersonalization (DP), and personal accomplishment (PA) were the three outcome measures. Two investigators independently extracted the data. The risk of bias was evaluated using Cochrane risk-of-bias tool for randomized trials (RoB2) and non-randomized studies of interventions (ROBINS-I). Cohen’s d and heterogeneity was estimated using a random-effects DerSimonian-Laird model and visualized by forest plots. Sensitivity analyses were carried out by leave-one-out meta-analysis.

**Results:**

We identified 33 eligible studies (*n* = 2536), comprising 25 (75.8%) individual intervention studies and 8 (24.2%) organizational intervention studies. Cohen’s d for individual intervention versus control were as follows: EE -0.25 (95% CI -0.40 to -0.11, *p* < 0.01, I^2^ = 49.3%), and DP -0.17 (95% CI -0.32 to -0.03, *p* = 0.02, I^2^ = 50.0%). The organizational intervention showed no significant association with any domain. Sensitivity analyses were robust in all outcomes, with differences in intervention description and design identified as potential contributors to heterogeneity.

**Conclusions:**

Various interventions, including individual coaching, meditation, and organization interventions, have been implemented to improve resident burnout. The effectiveness of intervention demonstrated none to small practical significance in improving burnout. Data inconsistency and high risk of bias across studies limited the validity of the pooled results. Further studies should focus on a combined approach.

**Registration:**

The study was registered on PROSPERO, under PROSPERO registration number CRD42022349698.

**Supplementary Information:**

The online version contains supplementary material available at 10.1186/s12909-024-06195-3.

## Introduction

Burnout syndrome, defined by the World Health Organization (WHO), is an occupational phenomenon from prolonged exposure to psychosocial risk factors in the work place [1], making it a serious [2–4] and prevalent [5–7] occupational health concern. This phenomenon, characterized by high emotional exhaustion (EE), high depersonalization (DP), and low personal accomplishment (PA), affects physicians and the patients they care for [6]. Its framework, largely influenced by Maslach [8–10], encompasses three domains. The ramifications of burnout are far-reaching: for providers, it can lead to mood disorders, family conflicts, diminished self-esteem, and early career departure; for patients, it associates with increased medical complications, legal challenges, prolonged hospital stay, and reduced satisfaction with healthcare [11–13]. In the United States alone, burnout is estimated to cost the healthcare sector $4.6 billion [3].

The residency period is widely recognized as one of the most stressful stages in a medical career, attributed to factors such as limited autonomy, high workloads, inadequate institutional support, and relatively low income [9, 14–16]. Previous studies, the systematic reviews and meta-analyses, have consistently highlighted the prevalence of burnout among resident physicians, with proportions ranging from 45 to 57% [5, 6, 17] globally. Consequently, over the past two decades, many training centers have initiated various interventions aimed at reducing burnout. These interventions encompass both individual-focused strategies, such as mindfulness training, meditation sessions, self-care courses, and psychological workshops [18–25], as well as organizational initiatives, including providing recreational opportunities, offering healthy food options, implementing rest days following shifts, adjusting shift schedules, and modifying shift duration [26–28].

Previous studies have frequently suggested a reduction in burnout syndrome following interventions targeted at resident physicians. However, there remains a lack of substantial evidence regarding the actual change in intervention effectiveness, which hinders the recommendations for the most suitable approaches. Given the importance of these interventions, a comprehensive and thorough review is essential. Therefore, this study aimed to evaluate the effectiveness of both individual and organizational interventions in reducing burnout among resident physician populations by conducting a systematic review and meta-analysis of existing evidence to assess their effectiveness.

## Materials and methods

### Eligibility criteria

To evaluate any intervention aimed at reducing burnout among resident physicians during their training, regardless of location or specialty. These interventions included randomized controlled trial (RCTs) or non-randomized studies of intervention. Accepted study designs encompassed concurrent non-randomized studies, pre-post studies, or historical control studies. Publications to be included can be in any languages regardless of the publication year, with available online full text. Measurement criteria for evaluation of interventions should utilized the Maslach Burnout Inventory (MBI) [10], with reporting on total scores of each dimension: EE, DP, and PA. Modification to the PROSPERO protocol was made to cover MBI scale other than the 22-item standard version in order to capture all literatures. For analytic purposes, interventions are pre-specified into either individual or organizational categories, the standard definition was derived from the documented types of stress management interventions (SMIs) by the Health and Safety Executive (HSE), United Kingdom [29].

### Exclusion criteria

Studies that focused solely on other healthcare personnel (such as nurses, pharmacists, dentists, medical students, and intern physicians), without providing subgroup data specifically for resident physicians and studies that were not available as full-text articles were excluded.

### Search strategies and data sources

The search was conducted across five databases: PubMed, Scopus, ScienceDirect, Embase, and the Cochrane Library, spanning December 1 to 21, 2023, with an updated search during the revision between August 19 and 26, 2024. The search process adhered to the PICO framework (Population, Intervention, Comparison, Outcome) and was executed by two investigators (WK and VS), following a stepwise syntax (See Supplementary Appendix 1, Additional File 1). Keywords and medical terms were derived from PubMed [30] and Cochrane Library MeSH (Medical Subject Headings) [31]. Duplicate records were managed using Endnote X9 software.

### Study selection

**Fig. 1:**
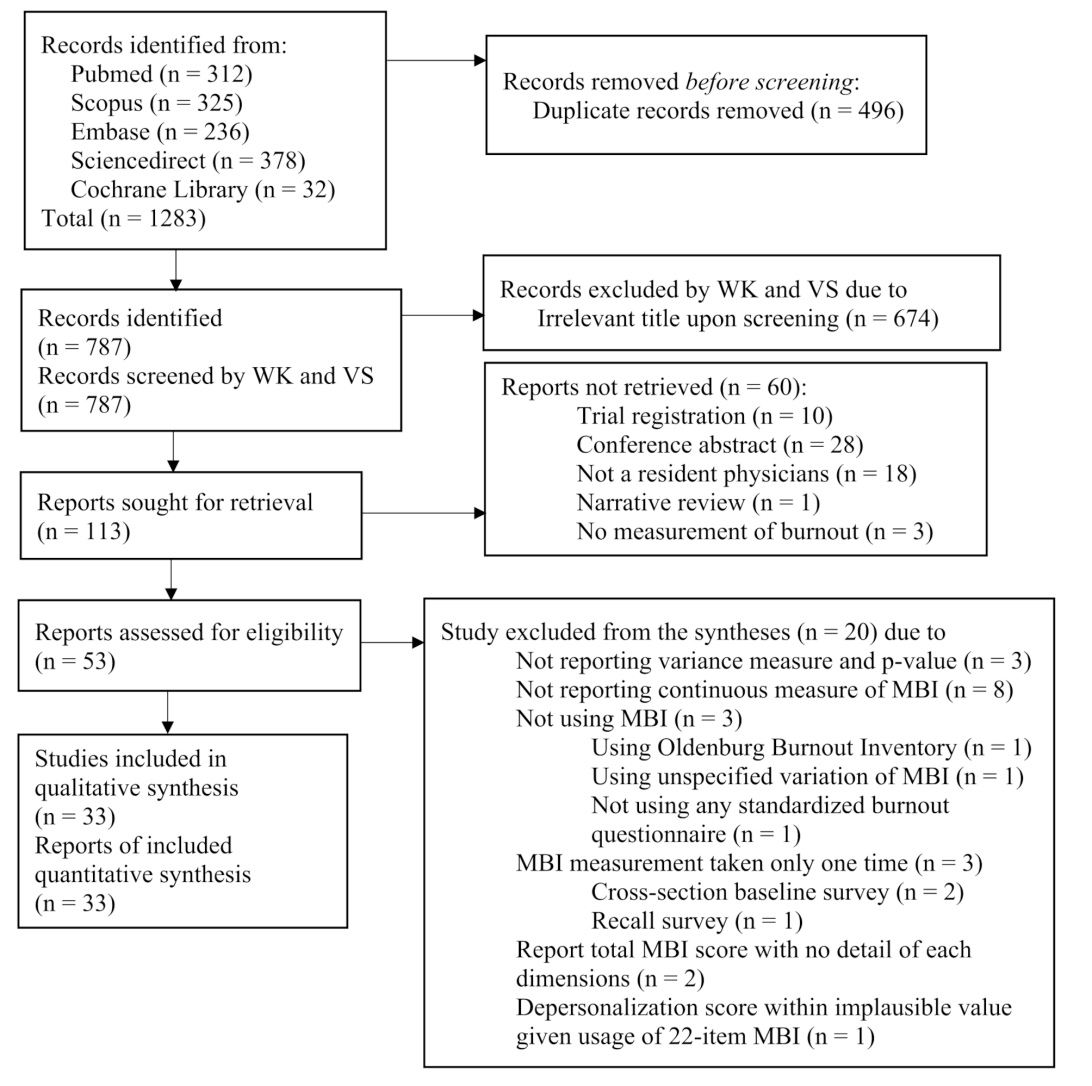
Preferred reporting items for systematic reviews and meta-analyses (PRISMA 2020) flow diagram of eligible studies

After two investigators (WK and VS) formulate the searching syntax together. These investigators then independently reviewed studies, excluding those without full texts or with irrelevant titles or abstracts. Then, the eligibility of each imported studies of each of the two investigators were deliberated upon, with consensus reached on eligible studies through discussion. In case of disagreement, a third investigator (WS) acted as an adjudicator. The screening process followed the PRISMA 2020 flow diagram (Fig. 1), PRISMA 2020 checklist (See Additional File 2) and PRISMA 2020 abstract checklist (See Additional File 3) [32] to ensure transparency and accuracy.

### Risk of bias assessment

Two reviewers (WK and VS) utilized the performed the RoB2 (Cochrane risk of bias assessment in randomized trial) [33] for randomized parallel studies and ROBINS-I (Risk of Bias in Non-randomized Studies-of Interventions) [34] for non-randomized studies to assess the risk of bias assessment. Independently, reviewers conducted these assessments between December 21 and 31, 2023. Then, during a discussion session on January 2, 2024, any disparities in findings were thoroughly discussed until a consensus was reached. Although a third investigator (WS) was available to adjudicate in case of disagreements, none arose during the process. Risk of bias assessment for additional studies was carried out during the revision between August 27 and 28, 2024.

### Data extraction

Two investigators (WK and VS) independently retrieved information from January 2 to 10, 2024, and updated upon revision from August 29, and 30, 2024. The extracted data for each study included the author’s name, country, year of publication, study design, intervention name, duration and frequency of sessions, study duration, participant count, specialty, and loss to follow-up. Additionally, outcome data concerning the mean and standard deviation in three domains of the Maslach Burnout Inventory–EE, DP, and PA–was collected at pre-intervention and post-intervention. In cases of incomplete outcome data, standard deviation was calculated from other reported metrics of comparison such as p-value, using the Cochrane Calculator [35, 36]. Furthermore, graphical data with no numerical description of data point were handled by PlotDigitizer.

### Data analyses

Analyses were done on STATA version 18.0 (StataCorp LLC, Texas, USA). Heterogeneity was assessed using Cochrane’s Q test and the I-squared statistics (I^2^) [37]. Due to the expected heterogeneity, the DerSimonian-Laird random-effects model was employed for meta-analysis [38]. Results were presented as post-intervention Cohen’s d standardized mean differences (SMD) and a 95% confidence interval, with visualization carried out by the forest plots. A two-sided p-value of < 0.05 was considered statistically significant. Sensitivity analyses were carried out with subgroup (See Supplementary Appendix 2.1 to 2.6, Additional File 1) and leave-one-out meta-analyses (See Supplementary Appendix 3.1 to 3.6, Additional File 1). Additionally, publication bias was explored by the funnel plots (See Supplementary Appendix 4.1 to 4.6, Additional File 1).

### Strength of evidence

Grading Quality of Evidence and Strength of Recommendations (GRADE) [39] approach was used to evaluate the strength of evidence for each outcome, separately for individual and organizational studies. Eight domains were assessed: inconsistency [40], indirectness [41], imprecision [42], risk of bias [33], publication bias, dose-response gradient, magnitude of association, and presence of residual confounding [43].

## Results

### Study selection and characteristics

We initially identified 1283 studies across five medical databases (See Supplementary Appendix 1, Additional File 1). After removing 496 redundant studies, 787 studies remained for screening. From this screening, 113 studies appeared potentially relevant based on their titles, leading to retrieval of the full paper. Ultimately, 53 studies met the criteria for inclusion as full-paper journal articles. Among them, 33 studies fulfilled the eligibility criteria [44–76]. No additional eligible studies were found through references searches. For a visual representation of the process, refer to the PRISMA 2020 flow diagram [32] (Fig. 1).

Table 1 presents the general characteristics of the thirty-three studies [44–76], including details such as author names, publication year, countries, baseline Maslach Burnout Inventory scores, medical specialties of participants, study designs, intervention descriptions, durations, and frequencies, outcome measurements, and loss to follow-up. Of these studies, 25 (75.8%) focused on individual interventions [50–72, 74, 75], while 8 (24.2%) addressed organizational interventions [44, 45, 47–49, 73, 76]. Among the individual interventions, 16 (64%) centered on coaching and emphasized aspects like self-development, resilience, and coping skills [59–72, 74], while 9 (36%) exclusively utilized meditation [50–58]. Regarding organizational intervention, 6 (75%) primarily targeted work-hour modification through changed in shift lengths and rest days after shift [44, 45, 47, 73, 76], while 2 (25%) focused on creating improved learning environment, such as healthy food delivery programs and workflow modifications [48, 49].

**Table 1:** Characteristics of thirty-three eligible studies

Author (year), country Measurement	Baseline MBI scores		*N* control/ intervention, (specialty)	Design	Intervention	Control
	Intervention Mean (SD)	Control Mean (SD)				
Individual coaching interventions (16 studies)						
Ares (2019), United States (9-item aMBI)	EE: 7.6 (3.1) DP: 6.5 (4.4) PA: 15.9 (3.3)	EE: 7.6 (3.1) DP: 6.5 (4.4) PA: 15.9 (3.3)	25/21 (Neurosurgery)	Historical-control	Mode: Bimonthly wellness lecture Duration: NA Frequency: 0.5 times per month Length: 12 months Dropout: 0 (0%)	Mode: Pre-intervention, previous academic year Dropout: 0 (0%)
Bragard (2008), Belgium (22-item MBI)	EE: 25.2 (9.2) DP: 9.2 (5.3) PA: 37.2 (5.6)	EE: 26.7 (8.4) DP: 9.1 (5.1) PA: 35.8 (5.5)	58/57 (Mixed)	Randomized controlled trial	Mode: 30-hour communication skills and 10-hour stress management skills Duration: 4 h per week Frequency: 10 times per month Length: 5 months Dropout: 9 (16%)	Mode: Waitlist control Dropout: 10 (18%)
Fainstad (2022), United States (22-item MBI)	EE: 26.0 (8.1) DP: 10.9 (5.5) PA: 35.8 (5.7)	EE: 28.2 (8.9) DP: 11.1 (5.6) PA: 33.7 (6.9)	50/51 (Mixed)	Randomized controlled trial	Mode: Online group-coaching program Duration: 1 h per week Frequency: 8 times per month Length: 6 months Dropout: 16 (32%)	Mode: Waitlist control Dropout: 6 (12%)
Hart (2019), United States (22-item MBI)	EE: 24.3 (9.8) DP: 14.2 (5.4) PA: 33.1 (5.0)	EE: 24.3 (9.8) DP: 14.2 (5.4) PA: 33.1 (5.0)	46/46 (Emergency medicine)	Self-control	Mode: Corporate wellness lectures Duration: 1 h per week Frequency: 1 times per month Length: 6 months Dropout: 22 (48%)	Mode: Pre-intervention baseline characteristics of the participants Dropout: 12 (26%)
Individual coaching interventions (16 studies), continued						
Huang (2020), China (22-item MBI)	EE: 16.4 (4.8) DP: 7.0 (3.4) PA: 28.5 (7.1)	EE: 15.8 (5.5) DP: 6.9 (2.8) PA: 28.1 (7.7)	18/18 (Mixed)	Randomized controlled trial	Mode: Balint group Duration: 1 h per week Frequency: 2 times per month Length: 6 months Dropout: 0 (0%)	Mode: Waitlist control Dropout: 0 (0%)
Martins (2011), Argentina (22-item MBI)	EE: 22.8 (7.4) DP: 7.3 (3.4) PA: 36.5 (3.5)	EE: 22.0 (6.4) DP: 6.7 (3.3) PA: 34.8 (3.7)	37/37 (Pediatrics)	Randomized controlled trial	Mode: Brief intervention Duration: 3 h per week Frequency: 2 times per month Length: 1 months Dropout: 0 (0%)	Mode: Waitlist control Dropout: 0 (0%)
Milstein (2012), United States (22-item MBI)	EE: 26.0 (6.6) DP: 9.1 (6.3) PA: 34.3 (5.9)	EE: 21.2 (10.1) DP: 12.0 (5.4) PA: 43.6 (3.5)	7/8 (Pediatrics)	Randomized controlled trial	Mode: Individual psychotherapeutic toll (brief intervention - BATHE technique) Duration: 0 h per week Frequency: 12 times per month Length: 3 months Dropout: 0 (0%)	Mode: Waitlist control Dropout: 0 (0%)
Palamara (2021), United States (22-item MBI)	EE: NA (8.8) DP: NA (8.4) PA: NA	EE: NA (8.8) DP: NA (8.4) PA: NA	235/235 (Internal medicine)	Self-control	Mode: Professional Development Coaching Program Duration: 1 h per week Frequency: 0.3 times per month Length: 8 months Dropout: 117 (50%)	Mode: Pre-intervention baseline characteristics of the participants Dropout: 117 (50%)
Individual coaching interventions (16 studies), continued						
Riall (2017), United States (16-item MBI)	EE: 16.8 (8.4) DP: 10.3 (7.9) PA: 27.8 (6.9)	EE: 16.8 (8.4) DP: 10.3 (7.9) PA: 27.8 (6.9)	49/49 (General surgery)	Self-control	Mode: Energy Leadership executive coaching model Duration: NA Frequency: 1 times per month Length: 12 months Dropout: 10 (20%)	Mode: Pre-intervention baseline characteristics of the participants Dropout: 10 (20%)
Sheer (2021), United States (22-item MBI)	EE: 10.6 (8.3) DP: 10.4 (8.0) PA: 38.5 (6.4)	EE: 10.6 (8.3) DP: 10.4 (8.0) PA: 38.5 (6.4)	107/107 (Internal medicine)	Self-control	Mode: Wellness morning reports by resident and discussion group by senior residents (Grassroot Interventions) Duration: 1 h per week Frequency: 2 times per month Length: 6 months Dropout: 65 (61%)	Mode: Pre-intervention baseline characteristics of the participants Dropout: 67 (63%)
Slavin (2016), United States (22-item MBI)	EE: 29.6 (9.3) DP: 10.2 (4.2) PA: NA	EE: 29.6 (9.3) DP: 10.2 (4.2) PA: NA	17/18 (Pediatrics)	Historical-control	Mode: Small workshop sessions targeted on stress management and life appreciation Duration: 1 h per week Frequency: 0.5 times per month Length: 12 months Dropout: 0 (0%)	Mode: Pre-intervention, previous academic year Dropout: 0 (0%)
Individual coaching interventions (16 studies), continued						
Song (2020), United States (9-item aMBI)	EE: 7.6 (4.2) DP: 5.2 (4.5) PA: 16.2 (1.8)	EE: 7.6 (4.2) DP: 5.2 (4.5) PA: 16.2 (1.8)	25/25 (General surgery)	Self-control	Mode: Resilience coaching program with workshops Duration: 1 h per week Frequency: 0.7 times per month Length: 8 months Dropout: 0 (0%)	Mode: Pre-intervention baseline characteristics of the participants Dropout: 0 (0%)
Seeland (2024), United States (22-item MBI)	EE: 25.5 (9.6) DP: 9.5 (4.3) PA: 39.7 (5.8)	EE: 25.5 (9.6) DP: 9.5 (4.3) PA: 39.7 (5.8)	58/58 (Obstetrics and gynecology)	Historical-control	Wellness Wednesday, wellness week, wellness workshops Duration: NA Frequency: 0.33 time per month Length: 24 months Dropout: 17 (35%)	Mode: Pre-intervention baseline characteristics of the participants Dropout: 20 (42%)
Stephanie (2022), Philippines (22-item MBI)	EE: 30.2 (10.0) DP: 13.7 (5.6) PA: 33.5 (4.9)	EE: 30.2 (10.0) DP: 13.7 (5.6) PA: 33.5 (4.9)	59/59 (Mixed)	Self-control	Mode: I-CARE program (communication skill workshops) Duration: NA Frequency: 2 times per month Length: 6 months Dropout: 42 (71%)	Mode: Pre-intervention baseline characteristics of the participants Dropout: 0 (0%)
Wild (2018), United States (22-item MBI, average score)	EE: 2.6 (1.5) DP: 2.4 (1.6) PA: 5.1 (1.1)	EE: 2.6 (1.5) DP: 2.4 (1.6) PA: 5.1 (1.1)	31/31 (Mixed)	Historical-control	Mode: Patient-centered communication training Duration: 1 h per week Frequency: 4 times per month Length: 36 months Dropout: 0 (0%)	Mode: Pre-intervention, previous academic year Dropout: 0 (0%)
Individual coaching interventions (16 studies), continued						
Winer (2019), United States (22-item MBI)	EE: 20.0 (9.4) DP: 13.0 (4.8) PA: 38.0 (3.4)	EE: 20.0 (9.4) DP: 13.0 (4.8) PA: 38.0 (3.4)	36/36 (General surgery)	Self-control	Mode: Comprehensive resident curriculum (This Week in SCORE) Duration: 1 h per week Frequency: 4 times per month Length: 12 months Dropout: 19 (53%)	Mode: Pre-intervention baseline characteristics of the participants Dropout: 19 (53%)
Individual meditation interventions (9 studies)						
Carullo (2021), United States (9-item aMBI)	EE: 9.9 (3.9) DP: 7.3 (4.3) PA: 13.4 (2.0)	EE: 9.9 (3.9) DP: 7.3 (4.3) PA: 13.4 (2.0)	53/53 (Anesthesiology)	Self-control	Mode: Smartphone meditation application Duration: 1 h per week Frequency: 30 times per month Length: 4 months Dropout: 22 (42%)	Mode: Pre-intervention baseline characteristics of the participants Dropout: 22 (42%)
Dunne (2019), United States (22-item MBI)	EE: 26.0 (4.0) DP: 9.4 (1.7) PA: 36.7 (7.0)	EE: 26.5 (5.2) DP: 8.8 (1.0) PA: 35.8 (8.8)	29/29 (Emergency medicine)	Randomized controlled trial	Mode: Attention-based training program (mantra meditation) Duration: 4 h per week Frequency: 2 times per month Length: 2 months Dropout: 12 (41%)	Mode: Waitlist control Dropout: 4 (14%)
Loewenthal (2021), United States (22-item MBI)	EE: 3.4 (1.2) DP: 2.8 (1.5) PA: NA	EE: 3.2 (1.9) DP: 3.1 (2.1) PA: NA	38/18 (Mixed)	Randomized controlled trial	Mode: RISE program (Mindfulness-Based Stress Reduction by Yoga) Duration: 1 h per week Frequency: 4 times per month Length: 2 months Dropout: 12 (32%)	Mode: Waitlist control Dropout: 2 (11%)
Individual meditation interventions (9 studies), continued						
Pandit (2022), United Kingdom (9-item aMBI)	EE: 7.5 (4.8) DP: 5.0 (1.2) PA: 15.0 (6.0)	EE: 7.5 (4.8) DP: 5.0 (1.2) PA: 15.0 (6.0)	21/21 (Neurosurgery)	Self-control	Mode: Mindfulness course Duration: 2 h per week Frequency: 4 times per month Length: 2 months Dropout: 0 (0%)	Mode: Pre-intervention baseline characteristics of the participants Dropout: 0 (0%)
Peterson (2021), United States (22-item MBI)	EE: 21.1 (12.2) DP: 8.3 (6.2) PA: 42.2 (3.4)	EE: 21.1 (12.2) DP: 8.3 (6.2) PA: 42.2 (3.4)	14/14 (Obstetrics and gynecology)	Self-control	Mode: Mindfulness course Duration: 2 h per week Frequency: 2 times per month Length: 3 months Dropout: 2 (14%)	Mode: Pre-intervention baseline characteristics of the participants Dropout: 0 (0%)
Purdie (2023), United States (9-item aMBI)	EE: 10.7 (4.8) DP: 6.0 (4.8) PA: 13.8 (3.9)	EE: 10.6 (4.0) DP: 5.9 (4.0) PA: 13.9 (3.1)	27/39 (Pediatrics)	Randomized controlled trial	Mode: Mindfulness Awareness Practices (MAPs) Duration: 2 h per week Frequency: 3 times per month Length: 1 months Dropout: 0 (0%)	Mode: Waitlist control Dropout: 0 (0%)
Schmeusser (2023), United States (22-item MBI)	EE: 14.5 (5.2) DP: 14.2 (7.2) PA: 37.7 (5.7)	EE: 14.5 (5.2) DP: 14.2 (7.2) PA: 37.7 (5.7)	24/24 (Obstetrics and gynecology)	Historical-control	Mode: Wellness program (meditation, guided reflection, and yoga) Duration and frequency: NA Length: 12 months Dropout: 6 (25%)	Mode: Pre-intervention, previous academic year Dropout: 5 (21%)
Individual meditation interventions (9 studies), continued						
Verweij (2017), Netherlands (20-item MBI)	EE: 16.5 (7.8) DP: 4.8 (3.0) PA: 32.8 (5.1)	EE: 14.5 (7.1) DP: 5.5 (3.9) PA: 32.9 (5.0)	80/68 (Mixed)	Randomized controlled trial	Mode: Mindfulness-Based Stress Reduction (MBSR) Duration: 3 h per week Frequency: 4 times per month Length: 2 months Dropout: 9 (11%)	Mode: Waitlist control Dropout: 1 (1%)
Weitzman (2021), United States (22-item MBI)	EE: NA (0.3) DP: NA (0.3) PA: NA (0.4)	EE: NA (0.3) DP: NA (0.3) PA: NA (0.4)	18/18 (Otolaryngology)	Self-control	Mode: Virtual reality meditation program Duration: 0 h per week Frequency: 1 times per month Length: 4 months Dropout: 0 (0%)	Mode: Pre-intervention baseline characteristics of the participants Dropout: 0 (0%)
Organizational work-hour interventions (6 studies)						
Burgos (2014), Argentina (22-item MBI)	EE: 29.0 (11.6) DP: 19.0 (12.3) PA: 31.0 (5.8)	EE: 29.0 (11.6) DP: 19.0 (12.3) PA: 31.0 (5.8)	25/25 (Cardiology)	Historical-control	Mode: Day of rest after shift Duration: NA Frequency: NA Length: 12 months Dropout: 2 (8%)	Mode: Pre-intervention, previous academic year Dropout: 6 (24%)
Parshuram (a) (2015), Canada (22-item MBI)	EE: 26.2 (11.0) DP: 13.0 (4.8) PA: 37.3 (4.9)	EE: 23.7 (10.2) DP: 9.8 (4.9) PA: 36.9 (7.4)	17/15 (Mixed)	Randomized controlled trial	Mode: Shift length modification from 24 to 12 h Duration: NA Frequency: NA Length: 2 months Dropout: 3 (18%)	Mode: 24-hour shift Length: 2 months Dropout: 2 (13%)
Organizational work-hour interventions (6 studies), continued						
Parshuram (b) (2015), Canada (22-item MBI)	EE: 26.4 (9.6) DP: 11.4 (7.4) PA: 35.3 (5.4)	EE: 23.7 (10.2) DP: 9.8 (4.9) PA: 36.9 (7.4)	15/15 (Mixed)	Randomized controlled trial	Mode: Shift length modification from 24 to 16 h Duration: NA Frequency: NA Length: 2 months Dropout: 1 (7%)	Mode: 24-hour shift Length: 2 months Dropout: 2 (13%)
Heppe (2024), United States (22-item MBI)	EE: 25 (IQR, 19–30) DP: 11 (IQR, 8–15) PA 38 (IQR, 33–41)	EE: 25 (IQR, 19–30) DP: 11 (IQR, 8–15) PA 38 (IQR, 33–41)	313/313 (Internal Medicine)	Historical-control	Mode: Alternate 4 + 4 block schedule (4 inpatient on-call weeks plus 4 outpatient off-call weeks) Duration: 24 months Frequency: NA Dropout: 97 (31%)	Mode: No alternate on-call and off-call schedule Duration: 24 months Frequency: NA Dropout: 97 (31%)
Schuh (2011), United States (22-item MBI)	EE: 23.3 (12.4) DP: 8.7 (6.6) PA: 35.6 (8.1)	EE: 23.3 (12.4) DP: 8.7 (6.6) PA: 35.6 (8.1)	34/34 (Neurology)	Self-control	Mode: Work hour limitation Duration: NA Frequency: NA Length: 1 months Dropout: 11 (32%)	Mode: Pre-intervention baseline characteristics of the participants Dropout: 10 (29%)
Stevens (2020), United States (22-item MBI)	EE: 2.7 (1.2) DP: 1.7 (0.9) PA: 4.6 (0.9)	EE: 2.7 (1.2) DP: 1.7 (0.9) PA: 4.6 (0.9)	19/19 (Otolaryngology)	Self-control	Mode: 2-hour protected nonclinical time Duration: 2 h per week Frequency: 4 times per month Length: 4 months Dropout: 0 (0%)	Mode: Pre-intervention baseline characteristics of the participants Dropout: 0 (0%)
Organizational improved learning environment interventions (2 studies)						
Bisgaard (2021), United States (22-item MBI)	EE: 23.5 (11.2) DP: 9.6 (4.4) PA: 32.8 (6.4)	EE: 23.5 (11.2) DP: 9.6 (4.4) PA: 32.8 (6.4)	59/59 (General surgery)	Historical-control	Mode: Healthy snacks delivery Duration: NA Frequency: 4 times per month Length: 24 months Dropout: 32 (54%)	Mode: Pre-intervention, previous academic year Dropout: 28 (47%)
Ogunyemi (2021), United States (22-item MBI)	EE: 28.1 (10.6) DP: 12.5 (6.6) PA: 38.5 (6.3)	EE: 28.1 (10.6) DP: 12.5 (6.6) PA: 38.5 (6.3)	130/130 (Mixed)	Historical-control	Mode: Learning environment and workflow streamlining Duration: NA Frequency: NA Length: 24 months Dropout: 9 (7%)	Mode: Pre-intervention, previous academic year Dropout: 0 (0%)

The majority of studies employed non-randomized, non-concurrent designs, with 9 (27.2%) using historical controls [44, 48, 49, 56, 59, 68, 71, 75, 76] and 13 (39.4%) utilizing self-control studies [47, 50, 53, 54, 58, 61, 65–67, 69, 70, 72, 73]. Eleven (33.3%) studies were randomized, controlled, concurrent trials [45, 51, 52, 55, 57, 60–64, 74]. Outcome measurements were conducted using various versions of the validated MBI. Specifically, 26 studies (78.7%) used the 22-item MBI [44, 45, 47–49, 51, 52, 54, 56, 58, 60–65, 67, 68, 70–76], 5 studies (15.2%) employed the 9-item MBI [50, 53, 55, 59, 69], 1 study (3.0%) used the 20-item Dutch version of the MBI [57], and 1 study (3.0%) utilized the 16-item MBI [66]. The median timeframe of interventions is 6 months (IQR, 3 to 12 months).

### Risk of bias in studies

According to Cochrane RoB2 [33], all randomized studies were rated as a high risk of bias (See Supplementary Appendix 5.1, Additional File 1). This bias primarily stemmed from the fourth domain, concerning subjective participant-reported outcomes without blinding. Moreover, with the exception of one study [60] (91.0%), there were issues with defining sequence generation and allocation concealment, resulting in a rating of some concerns regarding the first domain. Also, 8 studies [45, 51, 52, 57, 63, 64, 74], comprising 72.7% of the total, were categorized as high risk of bias in the second or third domain due to naive per protocol analysis from complete cases at the end of the studies.

All non-randomized studies were evaluated to be at high risk of bias using Cochrane ROBINS-I [34] (See Supplementary Appendix 5.2, Additional File 1), primarily due to inadequate confounder control, with historical control studies in particular. All studies were also susceptible to a high risk of bias arising from subjective participant-reported outcomes without blinding.

Leave-one-out sensitivity analyses demonstrated robustness across all outcome domains (See Supplementary Appendix 3.1 to 3.6, Additional File 1), with no suspected publication bias indicated by the funnel plots (See Supplementary Appendix 4.1 to 4.6, Additional File 1).

### Meta-analysis of individual intervention studies

**Fig. 2:**
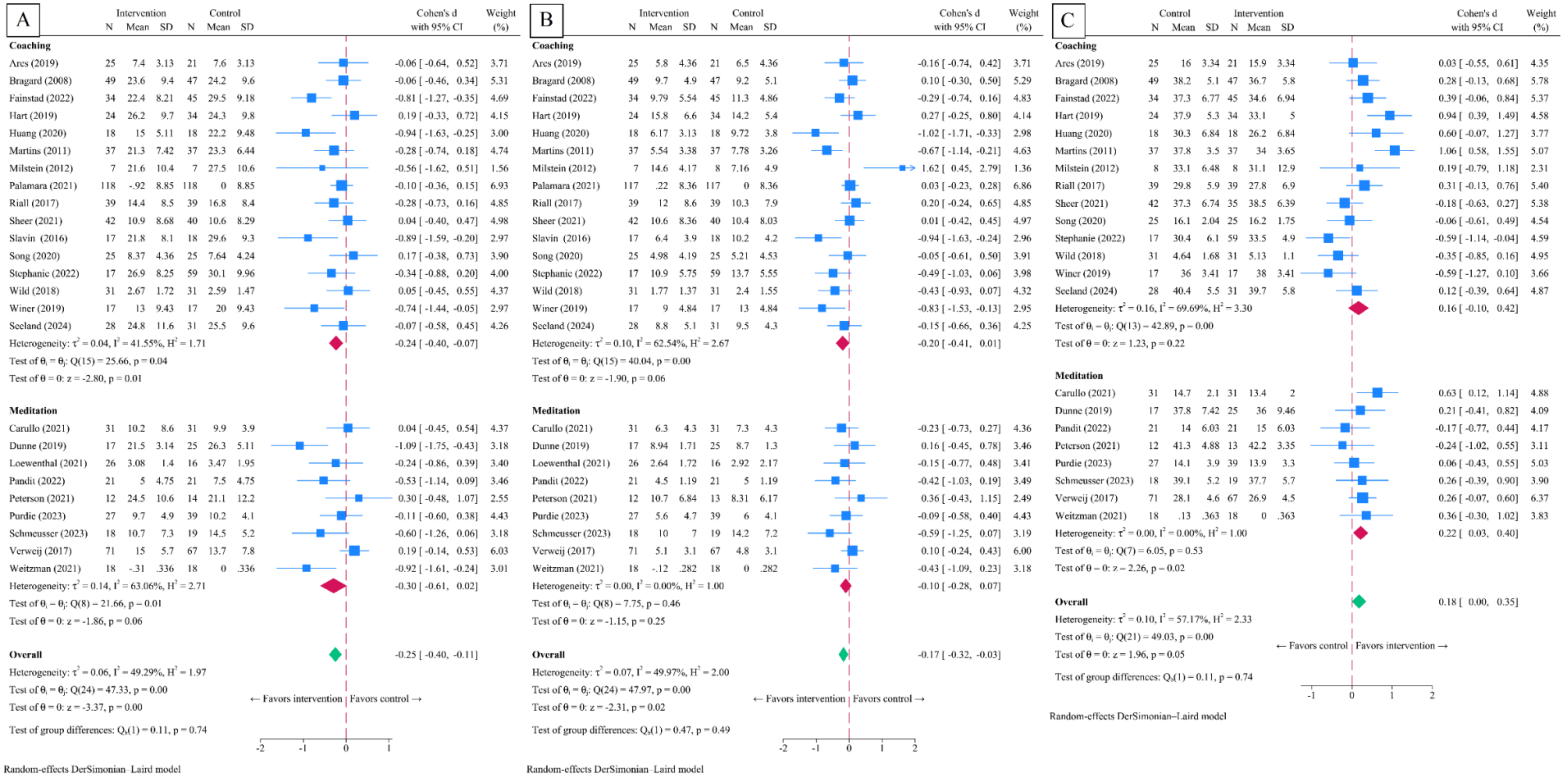
Post-intervention standardized mean difference in twenty-five individual interventions included in the systematic review and meta-analyses. Legends: panel **A**, emotional exhaustion; panel **B**, depersonalization; panel **C**, personal accomplishment

Comparison of the intervention group with the control group in individual intervention studies revealed a significant post-intervention Cohen’s d SMD in EE (-0.25, 95% CI -0.40 to -0.11, *p* < 0.001, I^2^ = 49.3%) (Fig. 2A) and DP (-0.18, 95% CI -0.32 to -0.03, *p* = 0.02, I^2^ = 50.0%) (Fig. 2B). However, there was no significant difference observed in PA (0.18, 95% CI 0.00 to 0.35, *p* = 0.05, I^2^ = 57.2%) (Fig. 2C).

Subgroup analyses of coaching intervention [59–72, 74, 75] demonstrated a post-intervention Cohen’s d SMD in EE (-0.24, 95% CI -0.40 to -0.07, *p* = 0.04, I^2^ = 41.6%). Nevertheless, non-significant differences were found for DP (-0.20, 95% CI -0.41 to 0.01, *p* = 0.07, I^2^ = 62.5%) and PA (0.16, 95% CI -0.10 to 0.42, *p* = 0.22, I^2^ = 69.7%). In the subgroup of meditation intervention studies [50–58], the Cohen’s d SMD was found to be non-statistically significant in EE (-0.30, 95% CI -0.61 to 0.02, *p* = 0.25, I^2^ = 63.1%) and DP (-0.10, 95% CI -0.28 to 0.07, *p* = 0.25, I^2^ = 0%), but statistically significant in PA (0.22, 95% CI 0.03 to 0.40, *p* = 0.02, I^2^ = 0%). Subgroup analyses for interventions with less than 6 months in timeframe yielded EE -0.32 (95% CI -0.61 to -0.03, *p* = 0.03, I^2^ = 58.3%), DP -0.12 (95% CI -0.40 to 0.15, *p* = 0.38, I^2^ = 56.2%), and PA (0.35, 95% CI 0.08 to 0.62, *p* = 0.01, I^2^ = 50.5%). Whereas in interventions with timeframe equals to 6 months and longer demonstrated EE -0.23 (95% CI -0.40 to -0.11, *p* = 0.01, I^2^ = 46.7%), and DP -0.19 (95% CI -0.38 to -0.02, *p* = 0.03, I^2^ = 49.5%), and PA (0.08, 95% CI -0.14 to 0.30, *p* = 0.47, I^2^ = 56.7%). (See Supplementary Appendix 2.1 to 2.3, Additional File 1).

### Meta-analysis of organizational intervention studies

**Fig. 3:**
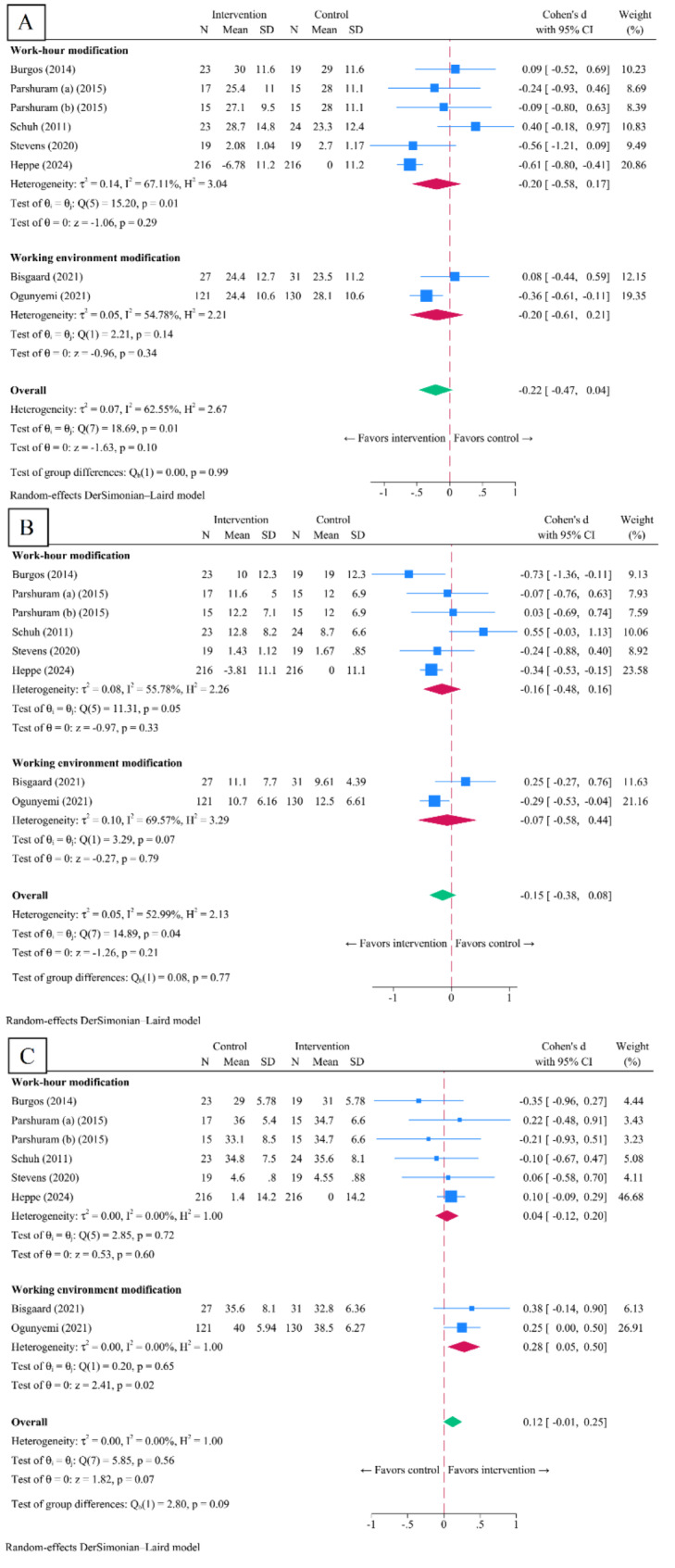
Post-intervention standardized mean score difference in eight organizational interventions included in the systematic review and meta-analyses. Legends: panel **A**, emotional exhaustion; panel **B**, depersonalization; panel **C**, personal accomplishment

In organizational intervention studies [44, 45, 47–49, 73, 76], pooling of post-intervention intervention Cohen’s d SMD yielded non-statistically significant resulted in all outcomes, EE (-0.22, 95% CI -0.47 to 0.04, *p* = 0.10, I^2^ = 62.6%) (Fig. 3A), DP (-0.15, 95% CI -0.38 to 0.08, *p* = 0.21, I^2^ = 53.0%) (Fig. 3B), and PA (0.12, 95% CI -0.01 to 0.25, *p* = 0.07; I^2^ = 0%) (Fig. 3C).

Subgroup analyses revealed no post-intervention Cohen’s d SMD in work-hour interventions [44, 45, 47, 73, 76] across all outcome domains: EE (-0.20, 95% CI -0.58 to 0.17, *p* = 0.29, I^2^ = 67.1%), DP (-0.16, 95% CI -0.48 to 0.16, *p* = 0.33, I^2^ = 55.8%), and PA (0.04, 95% CI -0.12 to 0.20, *p* = 0.60, I^2^ = 0%). Moreover, the subgroup of improved learning environment interventions [48, 49] showed non-statistically significant post-intervention mean score differences in EE (-0.20, 95% CI -0.61 to 0.21, *p* = 0.74, I^2^ = 54.8%) and DP (-0.07, 95% CI -0.58 to 0.44, *p* = 0.79, I^2^ = 69.6%), but a significant difference in PA (0.28, 95% CI 0.05 to 0.50, *p* = 0.02, I^2^ = 0%). Subgroup analyses for interventions with less than 6 months in timeframe yielded EE (0.07, 95% CI -0.32 to 0.46, *p* = 0.71, I^2^ = 6.9%), DP (0.22, 95% CI -0.18 to 0.61, *p* = 0.28, I^2^ = 8.2%), and PA (-0.04, 95% CI -0.42 to 0.34, *p* = 0.84, I^2^ = 0%). Whereas in interventions with timeframe equals to 6 months and longer demonstrated EE (-0.33, 95% CI -0.60 to -0.07, *p* = 0.01, I^2^ = 60.8%), DP -0.28 (95% CI -0.48 to -0.08, *p* = 0.01, I^2^ = 36.9%), and PA (0.14, 95% CI -0.01 to 0.29, *p* = 0.06, I^2^ = 6.8%). (See Supplementary Appendix 2.4 to 2.6, Additional File 1).

### GRADE evidence profile

All studies across different domains were predominantly non-randomized. Consequently, according to the GRADE evidence profile, we initially established low quality of evidence. However, due to the high risk of bias, we downgraded the quality assessment further, resulting in all studies providing very low quality of evidence (Table 2).

**Table 2:** GRADE evidence profile of thirty-three eligible studies

Outcomes	GRADE evidence profile*				Number of participants (studies)		Effect size (Cohen’s d)	Quality of the evidence (GRADE)
	Risk of bias	Inconsistency	Indirectness	Imprecision	Control group	Intervention group		
Individual coaching intervention compared to no intervention								
**Population**: Resident physicians **Setting**: Training center **Intervention**: Individual coaching intervention **Comparison**: No intervention								
Emotional exhaustion assessed with • 22-item MBI, with score ranging from 0 (low EE) to 54 (high EE) (*n* = 13) • 16-item MBI, with score ranging from 0 (low EE) to 30 (high EE) (*n* = 1) • 9-item aMBI, with score ranging from 0 (low EE) to 18 (high EE) (*n* = 2)	Serious	Serious	Not serious	Not serious	587 (5 historical-control, 6 self-control, 5 RCT studies)	528 (5 historical-control, 6 self-control, 5 RCT studies)	0.24 lower (0.40 lower to 0.07 lower)	Very low ⊕⊖⊖⊖
Depersonalization assessed with • 22-item MBI, with score ranging from 0 (low DP) to 30 (high DP) (*n* = 13) • 16-item MBI, with score ranging from 0 (low DP) to 30 (high DP) (*n* = 1) • 9-item aMBI, with score ranging from 0 (low DP) to 18 (high DP) (*n* = 2)	Serious	Serious	Not serious	Not serious	587 (5 historical-control, 6 self-control, 5 RCT studies)	528 (5 historical-control, 6 self-control, 5 RCT studies)	0.20 lower (0.41 lower to 0.01 higher)	Very low ⊕⊖⊖⊖
**Individual coaching intervention compared to no intervention**,** continued**								
Personal accomplishment assessed with • 22-item MBI, with score ranging from 0 (low PA) to 48 (high PA) (*n* = 11) • 16-item MBI, with score ranging from 0 (low PA) to 36 (high PA) (*n* = 1) • 9-item aMBI, with score ranging from 0 (low PA) to 18 (high PA) (*n* = 2)	Serious	Serious	Not serious	Not serious	451 (4 historical-control, 5 self-control, 5 RCT studies)	394 (4 historical-control, 5 self-control, 5 RCT studies)	0.16 higher (0.10 lower to 0.42 higher)	Very low ⊕⊖⊖⊖
**Individual meditation intervention compared to no intervention**								
**Population**: Resident physicians **Setting**: Training center **Intervention**: Individual meditation intervention **Comparison**: No intervention								
Emotional exhaustion assessed with • 22-item MBI, with score ranging from 0 (low EE) to 54 (high EE) (*n* = 5) • 20-item MBI with score ranging from 0 (low EE) to 48 (high EE) (*n* = 1) • 9-item aMBI, with score ranging from 0 (low EE) to 18 (high EE) (*n* = 3)	Serious	Not serious	Not serious	Not serious	250 (1 historical-control, 4 self-control, 4 RCT studies)	241 (1 historical-control, 4 self-control, 4 RCT studies)	0.33 lower (0.59 lower to 0.08 lower)	Very low ⊕⊖⊖⊖
**Individual meditation intervention compared to no intervention**,** continued**								
Depersonalization assessed with • 22-item MBI, with score ranging from 0 (low DP) to 30 (high DP) (*n* = 5) • 20-item MBI with score ranging from 0 (low DP) to 30 (high DP) (*n* = 1) • 9-item aMBI, with score ranging from 0 (low DP) to 18 (high DP) (*n* = 3)	Serious	Not serious	Not serious	Not serious	249 (1 historical-control, 4 self-control, 4 RCT studies)	241 (1 historical-control, 4 self-control, 4 RCT studies)	0.11 lower (0.34 lower to 0.11 higher)	Very low ⊕⊖⊖⊖
Personal accomplishment assessed with • 22-item MBI, with score ranging from 0 (low PA) to 48 (high PA) (*n* = 4) • 20-item MBI, with score ranging from 0 (low PA) to 42 (high PA) (*n* = 1) • 9-item aMBI, with score ranging from 0 (low PA) to 18 (high PA) (*n* = 3)	Serious	Not serious	Not serious	Not serious	233 (1 historical-control, 4 self-control, 3 RCT studies)	215 (1 historical-control, 4 self-control, 3 RCT studies)	0.21 higher (0.03 higher to 0.40 higher)	Very low ⊕⊖⊖⊖
**Organizational work-hour intervention compared to no intervention**								
**Population**: Resident physicians **Setting**: Training center **Intervention**: Organizational work-hour intervention **Comparison**: No intervention								
Emotional exhaustion assessed with • 22-item MBI, with score ranging from 0 (low EE) to 54 (high EE) (*n* = 6)	Serious	Serious	Not serious	Serious	308 (2 historical-control, 2 self-control, 2 RCT studies)	313 (2 historical-control, 2 self-control, 2 RCT studies)	0.20 lower (0.58 lower to 0.17 higher)	Very low ⊕⊖⊖⊖
**Organizational work-hour modification intervention compared to no intervention**,** continued**								
Depersonalization assessed with • 22-item MBI, with score ranging from 0 (low DP) to 30 (high DP) (*n* = 6)	Serious	Serious	Not serious	Serious	308 (2 historical-control, 2 self-control, 2 RCT studies)	313 (2 historical-control, 2 self-control, 2 RCT studies)	0.16 lower (0.49 lower to 0.16 higher)	Very low ⊕⊖⊖⊖
Personal accomplishment assessed with • 22-item MBI, with score ranging from 0 (low PA) to 48 (high PA) (*n* = 6)	Serious	Not serious	Not serious	Not serious	308 (2 historical-control, 2 self-control, 2 RCT studies)	313 (2 historical-control, 2 self-control, 2 RCT studies)	0.04 lower (0.12 lower to 0.20 higher)	Very low ⊕⊖⊖⊖
**Organizational improved learning environment compared to no intervention**								
**Population**: Resident physicians **Setting**: Training center **Intervention**: Organizational improved learning environment intervention **Comparison**: No intervention								
Emotional exhaustion assessed with • 22-item MBI, with score ranging from 0 (low EE) to 54 (high EE) (*n* = 2)	Serious	Serious	Not serious	Serious	161 (2 historical-control studies)	148 (2 historical-control studies)	0.20 lower (0.61 lower to 0.21 higher)	Very low ⊕⊖⊖⊖
Depersonalization assessed with • 22-item MBI, with score ranging from 0 (low DP) to 30 (high DP) (*n* = 2)	Serious	Serious	Not serious	Serious	161 (2 historical-control studies)	148 (2 historical-control studies)	0.07 lower (0.58 lower to 0.44 higher)	Very low ⊕⊖⊖⊖
Personal accomplishment assessed with • 22-item MBI, with score ranging from 0 (low PA) to 48 (high PA) (*n* = 2)	Serious	Not serious	Not serious	Not serious	161 (2 historical-control studies)	148 (2 historical-control studies)	0.28 higher (0.05 higher to 0.50 higher)	Very low ⊕⊖⊖⊖

*No studies fulfil the upward rating of evidence in large magnitude of an effect, dose-response gradient, and the effect of plausible residual confounding; no publication bias was found in all outcomes

## Discussion

To our knowledge, this systematic review and meta-analysis examined the effectiveness of interventions reducing burnout aimed at resident physicians, both at the individual and organizational level. Our findings indicate that individual interventions were significantly associated with reduced EE and DP scores, as measure by Cohen’s d SMD, compared with no interventions. However, it is important to note that according to the Cochrane Handbook of Meta-analysis [77], although statistically significant, the effect sizes observed were considered to have small practical significant. Furthermore, organizational interventions did not show any significant association with any domain of burnout.

Previous systematic reviews conducted on general practitioners (GP) and other health personnel yielded similar results to our findings. EE scores consistently reduced across all reporting studies [19, 78]. Some studies also showed a trend towards reduced DP scores [26], with a few demonstrating statistically significant results [19, 78]. However, the inconsistent in reduction in DP only reached statistically significance when pooling all individual interventions. This increased significance was due to the inclusion of additional studies in the last two years [53–56, 60, 65, 70], enhancing the statistical power and precision, thus establishing small effect sizes. Conversely, the limited addition of new organizational studies during this period prevented the attainment of statistically significance in DP reduction [49]. PA scores were reported in only a few studies [26, 79], with significant improvements observed. However, our finding showed only a trend towards statistical significance, even with the inclusion of newer studies [53–56, 60, 65, 70].

Exploratory subgroup analyses revealed notable differences between the effects of individual coaching and individual meditation interventions. In the case of individual coaching, post-intervention Cohen’s d SMD in EE scores were statistically significant, although with a small practical significance. Conversely, for individual meditation interventions, statistically significant were observed in PA scores, also with a small practical significance. This suggests distinct outcomes for these two types of interventions reducing burnout. This finding aligns with a recent clustered randomized study [80] conducted among a similar group of physicians. We suspected that various factors such as the characteristics of interventions, participant preferences, and voluntariness [81] might have influenced these results. Meditation sessions, focusing on breath and posture, differed significantly from the interactive, contemporary psychological techniques offered by coaching interventions in addressing day-to-day clinical demands. Consequently, they may have targeted distinct domain of burnout [82, 83]. While some studies suggest that coaching can help individuals discover and reflect on their strengths [84, 85], this effect was not clearly observed in our population, possibly due to differences in the content of each coaching intervention’s curriculum. In summary, our findings suggest the influence of interventions characteristics on the observed outcomes, as well as emphasizing the potential benefits of combining mediation with coaching interventions, may lead to improvement in both EE and PA [62, 66, 86]. This highlights the potential synergy between these approaches in addressing mitigating burnout among resident physicians.

In studies focusing on organizational intervention, improvement in PA scores were pronounced in interventions targeting improved learning environment compared to those addressing work hours. This difference may be attributed to the lessor disruption to personal schedules caused by interventions such as healthy food catering and workflow streamlining, as opposed to modifications to work hours. Changes in work hours can pose challenges to the continuity of patient care and shift transitions [45, 73, 87, 88]. Additionally, abrupt mandatory changes imposed by overseeing organizations may be perceived negatively by resident physicians, who may see them as a reduction in their already limited autonomy over work hours [73, 89]. This perception is supported by other systematic reviews on resident physicians and work-hour restrictions [90]. In summary, modifying work processes appears to better meet the needs of resident physicians compared to extensive changes to work hours [91]. This finding can help clarify the reasons behind the observed differences in PA score improvements between various organizational interventions. It emphasizes the potential challenges associated with modifying work hours and underscores the importance of considering resident physicians’ autonomy and needs when implementing interventions.

This review demonstrated several methodology strengths and adherence to recommendation guidelines outlined by Cochrane [77] and PRISMA [32] for a systematic review and meta-analysis. We utilized standardized quality assessment tools, namely RoB2 [33], ROBINS-I [34], and GRADE [39–43] to comprehensively evaluate risk of bias and certainty of evidences. Also, apart from individual and organizational intervention, we provided subgroup analyses to find possible differences in effect sizes across different study attributes. The robustness of sensitivity analyses and low risk of publication bias provided us with reliability and impartiality of the synthesized results. Additionally, in employing SMD, enables us to assess both statistical and practical significance. However, it is crucial to interpret the findings cautiously due to described limitations. Firstly, we included in our search strategy only the MBI as diagnostic tool for burnout. Different tools are nowadays existing for evaluate burnout. Examples included Melamed Burnout Questionnaire (SMBQ) [92], Oldenburg Burnout Inventory (OLBI) [93], Copenhagen Burnout Inventory (CBI) [94] and School Burnout Inventory (SBI) [95]. Secondly, conducting pairwise meta-analyses necessitated assuming comparability between control and intervention types, leading to significant heterogeneity, possibly stemming from methodological differences among intervention and control groups [37, 96, 97]. Population heterogeneity, including specialty types and cultural contexts, may also influence intervention effectiveness and compliance. High heterogeneity in outcome domains, often observed in other meta-analyses [18, 24, 26, 78, 98], suggests a mix of healthcare professionals in the studies [24], complicating the interpretation. Limited intervention comparability further contributed to heterogeneity [24–27, 78]. Thirdly, the included studies’ risk of bias was high, consistent with previous assessments [18, 24, 78], due to subjective participant-reported outcomes without blinding and inadequate confounder control in non-randomized studies [33]. Fourthly, organizational interventions were limited in varieties and numbers, which may result in underpower in detecting the true effect sizes. Therefore, any reported in burnout scores should be cautiously interpreted [20, 23, 79].

The implications of this study for practice and policy are substantial, particularly within postgraduate medical education curricula. Individual coaching interventions exhibit promise in reducing EE, with the potential for even greater impact when combined with meditation interventions to enhance PA. Individual coaching intervention consisted of positive psychology workshop such as resilience, stress management, and also encompassed the individual-driven development of soft skill include teamwork and communication. The qualitative synthesis of intervention characteristics, along with the quantitative synthesis consisting of subgroup analyses by implementation timeframe, provided an insight to the optimal intervention duration of longer than 6 months. Regarding those individual coaching interventions, we suggest 1–2 h per week in frequency of 1–2 times per month, with sustained activity for 6–12 months in order to harness their effectiveness. Whereas meditation should be practiced 1–2 h per week in frequency of 1–2 times per month for 6–12 months to properly introduce participants to its concept. On the other hand, the organizational interventions, especially those centered on work-hour modifications, have demonstrated limited benefits, while interventions addressing improved learning environment have shown improvement in PA. In this case, we recommended that work-hour modifications included shift-length modification, work-hour limitation and day-of-rest after shift should be evaluated after the participant have been able to adjust to the new work schedule, optimally 2–4 months after initiation. However, for more complex organizational interventions to improve learning environment include workflow streamlining and healthy snacks delivery should be evaluated in longer timeframe, in terms of 1–2 years for their effectiveness. Nevertheless, a critical consideration for program coordinators before implementing interventions is participant compliance, which requires careful planning and solutions. Finally, qualitative syntheses suggest considering a mixed bundle of approaches to burnout prevention, incorporating both individual and organizational interventions for synergistic effectiveness [18, 22–24, 26, 27].

For future studies, rigorous methodologies are essential to confirm the synthesized evidence. Randomized studies, such as preference-based trials [99], and non-randomized studies with targeted trial frameworks, incorporating adequate baseline and time-varying confounder control methods like regression and inverse probability weighting can enhance the effectiveness of outcomes [34, 100, 101]. Additionally, organizational interventions could be more efficiently using cluster parallel [102] or step-wedge design RCTs [103], which harness collective compliance within physician clusters in the same specialties. Alternatively, time-series designs may be suitable for organizational interventions [104] in institutes with active surveillance and consistent data collection of burnout, allowing for the assessment of long-term population-level changes in MBI scores [105, 106].

## Conclusions

A diverse array of interventions, both individual and organizational interventions, have been implemented among resident physicians. Individual coaching intervention led to a small yet significant improvement in EE, while individual meditation interventions were associated with a similar small but significant enhancement in PA. Organizational intervention, primarily focused on improved learning environment, resulted in small but significant enhancements in PA. However, the strength of these recommendations is relatively limited due risk of bias and inconsistency in the data. Further studies should prioritize a combined approach, integrating both individual and organizational interventions, with a rigorous methodology aimed at generating credible evidence for a synergistic approach to prevention burnout in post-graduate medical education.

## Electronic supplementary material

Below is the link to the electronic supplementary material.

Supplementary Material 1

Supplementary Material 2

Supplementary Material 3

## Data Availability

The datasets generated and analyzed during the current study are available in the Open Science Framework (OSF) repository; DOI: 10.17605/OSF.IO/3T5RB.

## Abbreviations

CBI: Copenhagen Burnout Inventory
DP: Depersonalization
EE: Emotional exhaustion
GP: General practitioners
GRADE: Grading of Recommendations, Assessment, Development, and Evaluations
HSE: Health and Safety Executive
MBI: Maslach Burnout Inventory
MeSH: Medical Subject Headings
OLBI: Oldenburg Burnout Inventory
PA: Personal accomplishment
PICO: Participant, intervention, control, outcome
PRISMA: Preferred Reporting Items for Systematic reviews and Meta-Analyses
PROSPERO: International Prospective Register of Systematic Reviews
RCT: Randomized-controlled trial
RoB2: Cochrane Risk-of-Bias Tool for Randomized Trials
ROBINS-I: Cochrane Risk Of Bias In Non-randomized Studies - of Interventions
SBI: School Burnout Inventory
SMBQ: Shirom-Melamed Burnout Questionnaire
SMD: Standardized mean difference
SMIs: Stress Management Interventions
WHO: World Health Organization
