# Supplementary material for: Individual and organizational interventions to reduce burnout in resident physicians: a systematic review and meta-analysis
Source: BMC Med Educ. 2024 Oct 30;24:1234. doi: 10.1186/s12909-024-06195-3 (PMC11523819; doi:10.1186/s12909-024-06195-3)

Additional File 1.

Individual and Organizational Interventions to Reduce Burnout in Resident Physicians: A Systematic Review and Meta-analysis

1. **Supplementary Appendix 1.** Searching syntax from (A) PubMed, (B) Embase, (C) Cochrane Library, (D) Scopus, (E) Sciencedirect
2. **Supplementary Appendix 2.1.** Subgroup meta-analysis of post-intervention standardized mean difference of EE score in individual intervention studies
3. **Supplementary Appendix 2.2.** Subgroup meta-analysis of post-intervention standardized mean difference of DP score in individual intervention studies
4. **Supplementary Appendix 2.3.** Subgroup meta-analysis of post-intervention standardized mean difference of PA score in individual intervention studies
5. **Supplementary Appendix 2.4.** Subgroup meta-analysis of post-intervention standardized mean difference of EE score in organizational intervention studies
6. **Supplementary Appendix 2.5.** Subgroup meta-analysis of post-intervention standardized mean difference of DP score in organizational intervention studies
7. **Supplementary Appendix 2.6.** Subgroup meta-analysis of post-intervention standardized mean difference of PA score in organizational intervention studies
8. **Supplementary Appendix 3.1.** Sensitivity analyses by leave-one-out method for standardized mean difference of EE score in individual intervention studies
9. **Supplementary Appendix 3.2.** Sensitivity analyses by leave-one-out method for standardized mean difference of DP score in individual intervention studies
10. **Supplementary Appendix 3.3.** Sensitivity analyses by leave-one-out method for standardized mean difference of PA score in individual intervention studies
11. **Supplementary Appendix 3.4.** Sensitivity analyses by leave-one-out method for standardized mean difference of EE score in organizational intervention studies
12. **Supplementary Appendix 3.5.** Sensitivity analyses by leave-one-out method for standardized mean difference of DP score in organizational intervention studies
13. **Supplementary Appendix 3.6.** Sensitivity analyses by leave-one-out method for standardized mean difference of PA score in organizational intervention studies
14. **Supplementary Appendix 4.1.** Funnel plot of standardized mean difference of EE score in individual intervention studies
15. **Supplementary Appendix 4.2.** Funnel plot of standardized mean difference of DP score in individual intervention studies
16. **Supplementary Appendix 4.3.** Funnel plot of standardized mean difference of PA score in individual intervention studies
17. **Supplementary Appendix 4.4.** Funnel plot of standardized mean difference of EE score in organizational intervention studies
18. **Supplementary Appendix 4.5.** Funnel plot of standardized mean difference of DP score in organizational intervention studies
19. **Supplementary Appendix 4.6.** Funnel plot of standardized mean difference of PA score in organizational intervention studies
20. **Supplementary Appendix 5.1.** Summary of the risk of bias assessment by Cochrane's RoB2 across 11 randomized controlled concurrent studies
21. **Supplementary Appendix 5.2.** Summary of the risk of bias assessment by Cochrane's ROBINS-I across 20 non-randomized studies

Supplementary Appendix 1. Searching syntax from (A) PubMed, (B) Embase, (C) Cochrane Library, (D) Scopus, (E) Sciencedirect

Database	Step	Search algorithm	Items found (August 26, 2024)
(A) Pubmed			
Population	#1	(residen* OR (residen* AND train*)) AND physician*	47603
Outcome	#2	burnout*	30030
	#3	psycho* AND burnout*	21659
	#4	#1 AND (#2 OR #3)	1385
	#5	mbi	5051
	#6	maslach burnout inventor*	3750
	#7	#5 OR #6	7368
	#8	#4 AND #7	312
(B) Embase			
Population	#1	(residen* OR (residen* AND train*)) AND physician*	65555
Outcome	#2	burnout*	39909
	#3	psycho* AND burnout*	20516
	#4	#1 AND (#2 OR #3)	2132
	#5	mbi*	31681
	#6	Maslach AND burnout AND inventor*	5416
	#7	#5 OR #6	39909
	#8	#4 AND #7	717
Type of studies	#9	#8 AND ('cohort analysis'/de OR 'comparative study'/de OR 'controlled study'/de OR 'human experiment'/de OR 'prospective study'/de OR 'randomized controlled trial'/de)	236
Intervention and control	#10	#9	236
(C) Cochrane Library			
Population	#1	((residen* OR (residen* AND train*)) AND physician*)	2495
Outcome	#2	Burnout*	1862
	#3	Mbi*	1502
	#4	Maslach AND burnout AND inventor*	336
	#5	(#3 OR #4)	1694
	#6	(#1 AND #5) AND trials	32

Database	Step	Search algorithm	Items found
(D) Scopus			
	#1	(residen* OR (residen* AND train*)) AND physician*	48582
	#2	burnout*	56756
	#3	psycho*	26501
	#4	#2 OR #3	26501
	#5	mbi*	18967
	#6	Maslach burnout inventor*	5997
	#7	#5 OR #6	6036
	#8	#1 AND (#4 OR #7)	361
	#9	#8 AND "Limited to Article"	325
(E) Sciencedirect			
	#1	resident	786408
	#2	burnout	55959
	#3	MBI	18117
	#4	Maslach burnout inventory	3929
	#5	#1 AND #2 AND (#3 OR #4)	1184
	#6	#5 AND research articles	700
	#7	#6 AND medicine and dentistry	589
	#8	#7 AND intervention	378

Supplementary Appendix 2.1. Subgroup meta-analysis of post-intervention standardized mean difference of EE score in individual intervention studies

Study subgroup	K	Cohen's d with 95% CI	p-value
**Subtypes**			
Coaching	16	-0.24 [-0.40, -0.07]	0.005
Meditation	9	-0.30 [-0.61, 0.02]	0.063
Test of group differences: $Q_b(1) = 0.11$, $p = 0.74$			
**Country of origin**			
Non-USA	6	-0.25 [-0.55, 0.05]	0.098
USA	19	-0.26 [-0.43, -0.08]	0.004
Test of group differences: $Q_b(1) = 0.00$, $p = 0.98$			
**Study design**			
Historical-control	5	-0.26 [-0.59, 0.07]	0.126
Self-control	11	-0.16 [-0.35, 0.03]	0.098
Randomized controlled trial	9	-0.38 [-0.68, -0.08]	0.014
Test of group differences: $Q_b(2) = 1.45$, $p = 0.48$			
**Measurement of outcome**			
22-item MBI	18	-0.34 [-0.53, -0.16]	0.000
9-item aMBI	5	-0.08 [-0.32, 0.17]	0.534
Other MBI variants	2	-0.02 [-0.49, 0.44]	0.924
Test of group differences: $Q_b(2) = 3.70$, $p = 0.16$			
**Specialty of participants**			
Single specialty	19	-0.25 [-0.41, -0.09]	0.002
Mixed specialty	6	-0.26 [-0.63, 0.10]	0.161
Test of group differences: $Q_b(1) = 0.00$, $p = 0.96$			
**Timeframe**			
<6 months	9	-0.32 [-0.61, -0.03]	0.030
≥6 months	16	-0.23 [-0.40, -0.05]	0.011
Test of group differences: $Q_b(1) = 0.29$, $p = 0.59$			
**Overall**		-0.25 [-0.40, -0.11]	0.001
Heterogeneity: $\tau^2 = 0.06$, $I^2 = 49.29\%$, $H^2 = 1.97$			
Test of $\theta_i = \theta_j$: $Q(24) = 47.33$, $p = 0.00$			

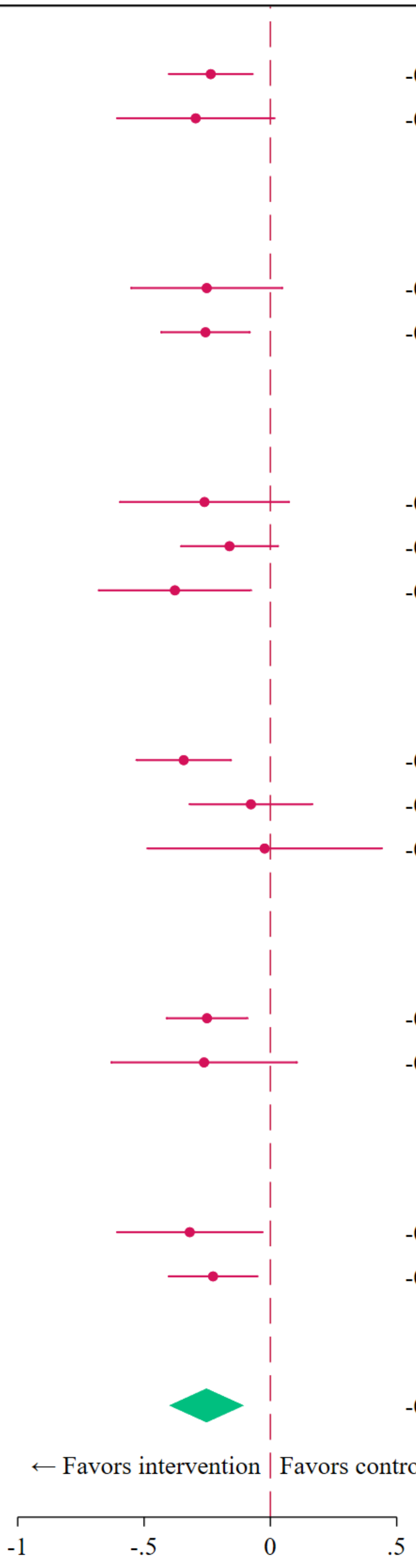

Random-effects DerSimonian–Laird model

Supplementary Appendix 2.2. Subgroup meta-analysis of post-intervention standardized mean difference of DP score in individual intervention studies

Study subgroup	K	Cohen's d with 95% CI	p-value
**Subtypes**			
Coaching	16	-0.20 [-0.41, 0.01]	0.058
Meditation	9	-0.10 [-0.28, 0.07]	0.251
Test of group differences: $Q_b(1) = 0.47$, p = 0.49			
**Country of origin**			
Non-USA	6	-0.35 [-0.70, 0.01]	0.055
USA	19	-0.12 [-0.28, 0.04]	0.141
Test of group differences: $Q_b(1) = 1.28$, p = 0.26			
**Study design**			
Historical-control	5	-0.40 [-0.67, -0.14]	0.003
Self-control	11	-0.10 [-0.28, 0.08]	0.290
Randomized controlled trial	9	-0.12 [-0.42, 0.19]	0.449
Test of group differences: $Q_b(2) = 3.62$, p = 0.16			
**Measurement of outcome**			
22-item MBI	18	-0.22 [-0.43, -0.02]	0.028
9-item aMBI	5	-0.18 [-0.42, 0.06]	0.144
Other MBI variants	2	0.14 [-0.13, 0.40]	0.320
Test of group differences: $Q_b(2) = 4.80$, p = 0.09			
**Specialty of participants**			
Single specialty	19	-0.16 [-0.34, 0.02]	0.076
Mixed specialty	6	-0.21 [-0.51, 0.08]	0.149
Test of group differences: $Q_b(1) = 0.09$, p = 0.76			
**Timeframe**			
<6 months	9	-0.12 [-0.40, 0.15]	0.381
≥6 months	16	-0.20 [-0.38, -0.02]	0.033
Test of group differences: $Q_b(1) = 0.18$, p = 0.67			
**Overall**		-0.17 [-0.32, -0.03]	0.021
Heterogeneity: $\tau^2 = 0.07$, $I^2 = 49.97\%$, $H^2 = 2.00$			
Test of $\theta_i = \theta_j$: $Q(24) = 47.97$, p = 0.00			

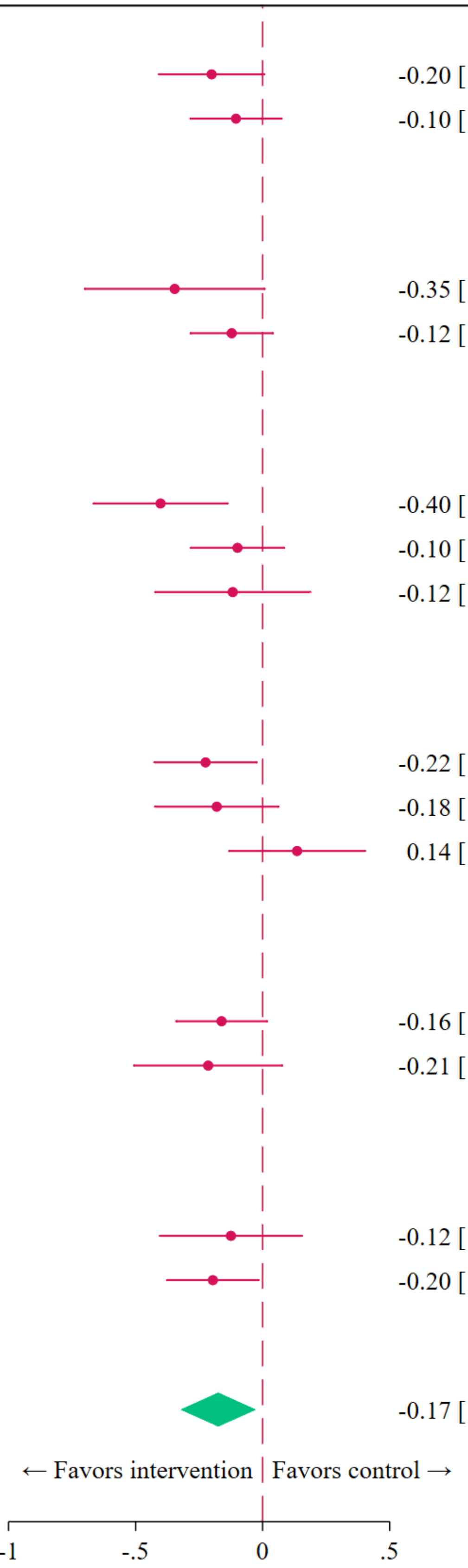

Random-effects DerSimonian–Laird model

Supplementary Appendix 2.3. Subgroup meta-analysis of post-intervention standardized mean difference of PA score in individual intervention studies

Study subgroup	K	Cohen's d with 95% CI	p-value
**Subtypes**			
Coaching	14	0.16 [-0.10, 0.42]	0.218
Meditation	8	0.22 [0.03, 0.40]	0.024
Test of group differences: $Q_b(1) = 0.11$, $p = 0.74$			
**Country of origin**			
Non-USA	6	0.25 [-0.17, 0.67]	0.248
USA	16	0.15 [-0.04, 0.33]	0.120
Test of group differences: $Q_b(1) = 0.19$, $p = 0.66$			
**Study design**			
Historical-control	4	-0.02 [-0.29, 0.26]	0.914
Self-control	10	0.06 [-0.25, 0.37]	0.706
Randomized controlled trial	8	0.38 [0.16, 0.60]	0.001
Test of group differences: $Q_b(2) = 5.71$, $p = 0.06$			
**Measurement of outcome**			
22-item MBI	15	0.18 [-0.08, 0.43]	0.181
9-item aMBI	5	0.12 [-0.16, 0.40]	0.408
Other MBI variants	2	0.28 [0.01, 0.55]	0.040
Test of group differences: $Q_b(2) = 0.70$, $p = 0.70$			
**Specialty of participants**			
Single specialty	17	0.15 [-0.07, 0.38]	0.179
Mixed specialty	5	0.22 [-0.04, 0.49]	0.095
Test of group differences: $Q_b(1) = 0.16$, $p = 0.69$			
**Timeframe**			
<6 months	8	0.35 [0.08, 0.62]	0.013
≥6 months	14	0.08 [-0.14, 0.30]	0.472
Test of group differences: $Q_b(1) = 2.26$, $p = 0.13$			
**Overall**		0.18 [0.00, 0.35]	0.050
Heterogeneity: $\tau^2 = 0.10$, $I^2 = 57.17\%$, $H^2 = 2.33$			
Test of $\theta_i = \theta_j$: $Q(21) = 49.03$, $p = 0.00$			

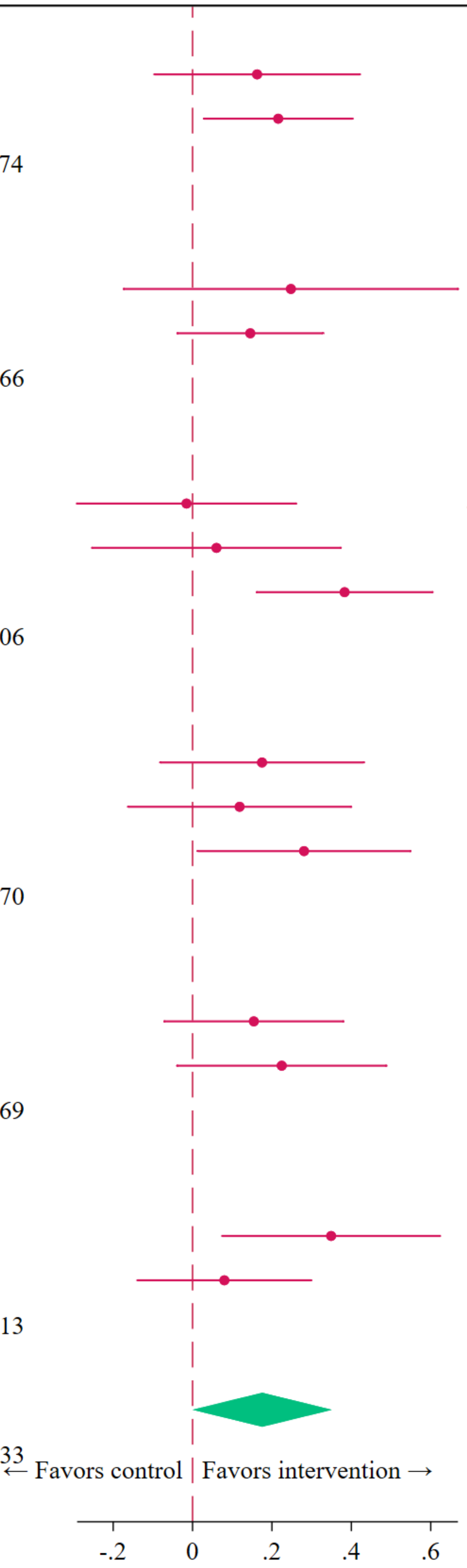

Random-effects DerSimonian–Laird model

Supplementary Appendix 2.4. Subgroup meta-analysis of post-intervention standardized mean difference of EE score in organizational intervention studies

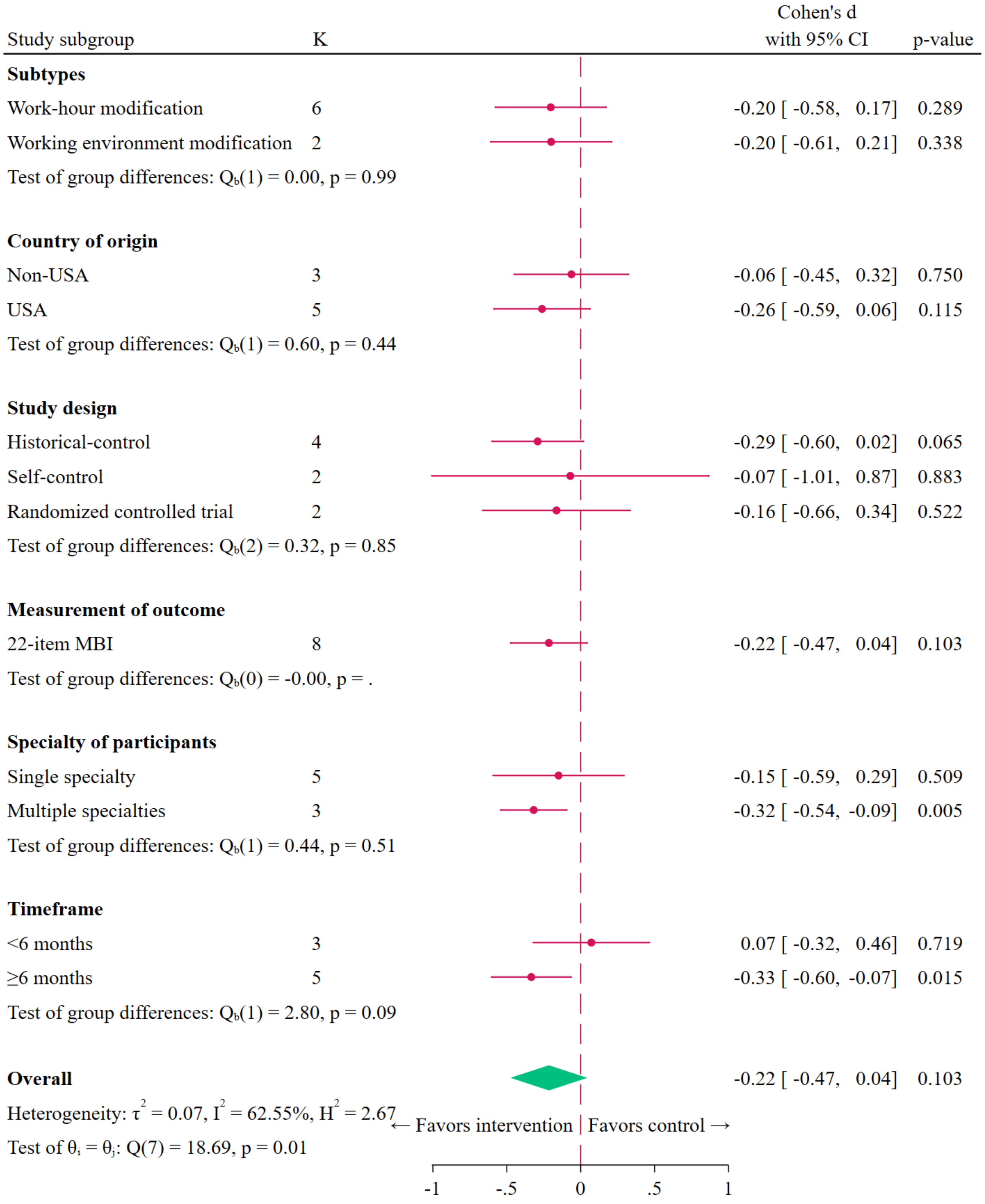

Random-effects DerSimonian–Laird model

Supplementary Appendix 2.5. Subgroup meta-analysis of post-intervention standardized mean difference of DP score in organizational intervention studies

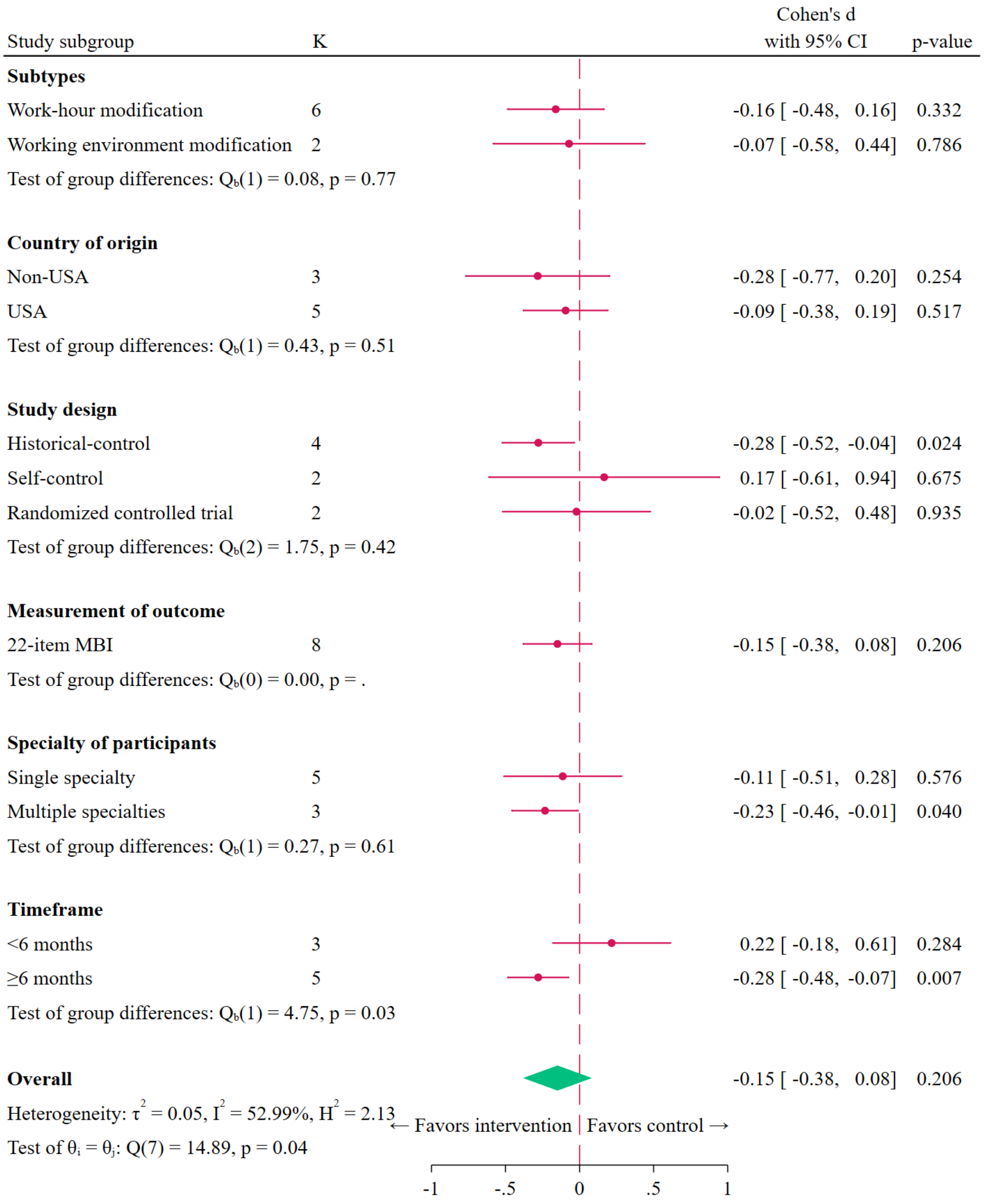

Random-effects DerSimonian–Laird model

Supplementary Appendix 2.6. Subgroup meta-analysis of post-intervention standardized mean difference of PA score in organizational intervention studies

Study subgroup	K	Cohen's d with 95% CI	p-value
**Subtypes**			
Work-hour modification	6	0.04 [-0.12, 0.20]	0.598
Working environment modification	2	0.28 [0.05, 0.50]	0.016
Test of group differences: $Q_b(1) = 2.80$, $p = 0.09$			
**Country of origin**			
Non-USA	3	-0.13 [-0.52, 0.25]	0.502
USA	5	0.15 [0.01, 0.29]	0.030
Test of group differences: $Q_b(1) = 1.83$, $p = 0.18$			
**Study design**			
Historical-control	4	0.15 [-0.04, 0.33]	0.130
Self-control	2	-0.03 [-0.46, 0.40]	0.890
Randomized controlled trial	2	0.01 [-0.49, 0.51]	0.969
Test of group differences: $Q_b(2) = 0.70$, $p = 0.70$			
**Measurement of outcome**			
22-item MBI	8	0.12 [-0.01, 0.25]	0.069
Test of group differences: $Q_b(0) = 0.00$, p = .			
**Specialty of participants**			
Single specialty	5	0.08 [-0.08, 0.24]	0.339
Multiple specialties	3	0.20 [-0.02, 0.43]	0.073
Test of group differences: $Q_b(1) = 0.83$, $p = 0.36$			
**Timeframe**			
<6 months	3	-0.04 [-0.42, 0.34]	0.840
≥6 months	5	0.14 [-0.01, 0.29]	0.063
Test of group differences: $Q_b(1) = 0.76$, $p = 0.38$			
**Overall**		0.12 [-0.01, 0.25]	0.069
Heterogeneity: $\tau^2 = 0.00$, $I^2 = 0.00\%$, $H^2 = 1.00$			
Test of $\theta_i = \theta_j$: $Q(7) = 5.85$, $p = 0.56$			

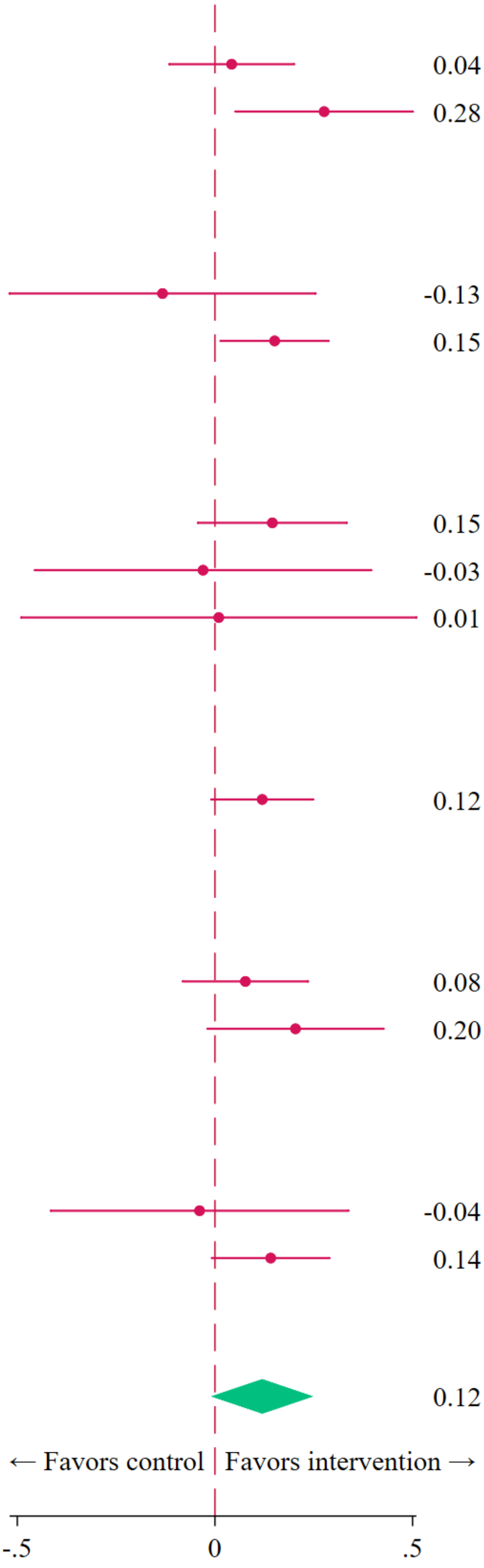

Random-effects DerSimonian–Laird model

Supplementary Appendix 3.1. Sensitivity analyses by leave-one-out method for standardized mean difference of EE score in individual intervention studies

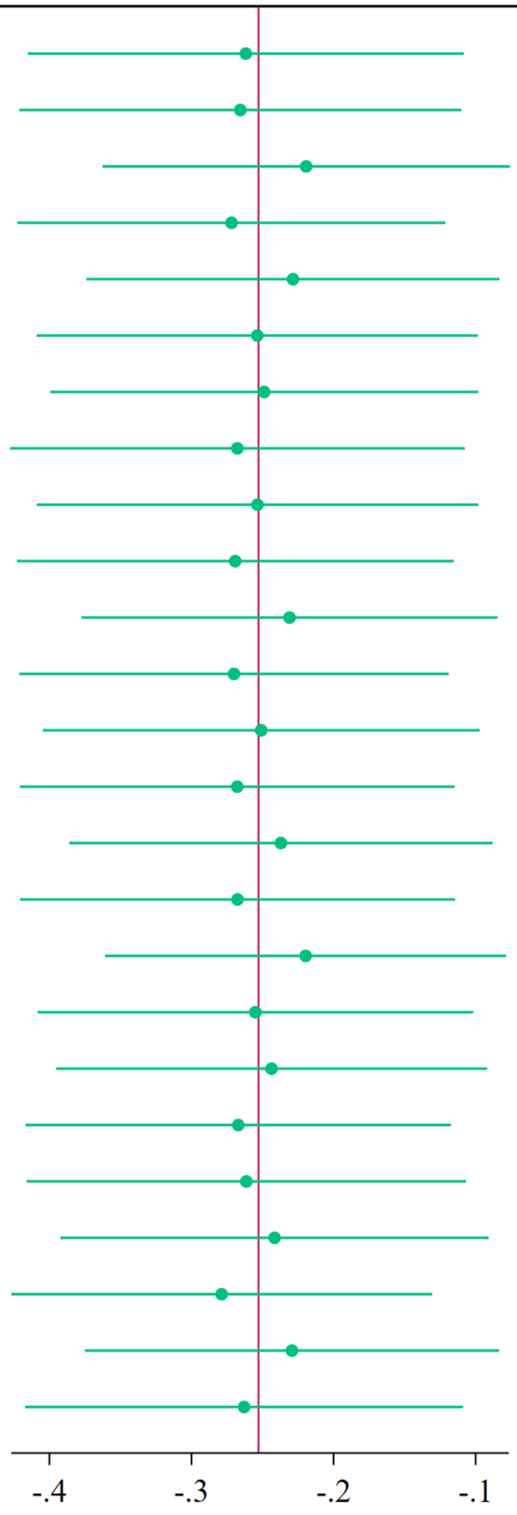

Omitted study	Cohen's d with 95% CI	p-value
Ares (2019)	-0.26 [-0.41, -0.11]	0.001
Bragard (2008)	-0.27 [-0.42, -0.11]	0.001
Fainstad (2022)	-0.22 [-0.36, -0.08]	0.003
Hart (2019)	-0.27 [-0.42, -0.12]	0.000
Huang (2020)	-0.23 [-0.37, -0.08]	0.002
Martins (2011)	-0.25 [-0.41, -0.10]	0.001
Milstein (2012)	-0.25 [-0.40, -0.10]	0.001
Palamara (2021)	-0.27 [-0.43, -0.11]	0.001
Riall (2017)	-0.25 [-0.41, -0.10]	0.001
Sheer (2021)	-0.27 [-0.42, -0.12]	0.001
Slavin (2016)	-0.23 [-0.38, -0.09]	0.002
Song (2020)	-0.27 [-0.42, -0.12]	0.000
Stephanie (2022)	-0.25 [-0.40, -0.10]	0.001
Wild (2018)	-0.27 [-0.42, -0.12]	0.001
Winer (2019)	-0.24 [-0.39, -0.09]	0.002
Carullo (2021)	-0.27 [-0.42, -0.12]	0.001
Dunne (2019)	-0.22 [-0.36, -0.08]	0.002
Loewenthal (2021)	-0.26 [-0.41, -0.10]	0.001
Pandit (2022)	-0.24 [-0.39, -0.09]	0.002
Peterson (2021)	-0.27 [-0.42, -0.12]	0.000
Purdie (2023)	-0.26 [-0.42, -0.11]	0.001
Schmeusser (2023)	-0.24 [-0.39, -0.09]	0.002
Verweij (2017)	-0.28 [-0.43, -0.13]	0.000
Weitzman (2021)	-0.23 [-0.37, -0.08]	0.002
Seeland (2024)	-0.26 [-0.42, -0.11]	0.001

Random-effects DerSimonian–Laird model

Supplementary Appendix 3.2. Sensitivity analyses by leave-one-out method for standardized mean difference of DP score in individual intervention studies

Omitted study	Cohen's d with 95% CI	p-value
Ares (2019)	-0.18 [-0.33, -0.02]	0.025
Bragard (2008)	-0.19 [-0.34, -0.04]	0.015
Fainstad (2022)	-0.17 [-0.32, -0.02]	0.031
Hart (2019)	-0.19 [-0.34, -0.04]	0.012
Huang (2020)	-0.15 [-0.29, -0.00]	0.043
Martins (2011)	-0.15 [-0.30, -0.00]	0.046
Milstein (2012)	-0.19 [-0.33, -0.06]	0.006
Palamara (2021)	-0.19 [-0.35, -0.03]	0.018
Riall (2017)	-0.19 [-0.35, -0.04]	0.012
Sheer (2021)	-0.19 [-0.34, -0.03]	0.019
Slavin (2016)	-0.15 [-0.29, -0.01]	0.041
Song (2020)	-0.18 [-0.33, -0.03]	0.021
Stephanie (2022)	-0.16 [-0.31, -0.01]	0.036
Wild (2018)	-0.16 [-0.32, -0.01]	0.035
Winer (2019)	-0.15 [-0.30, -0.01]	0.039
Carullo (2021)	-0.17 [-0.33, -0.02]	0.028
Dunne (2019)	-0.19 [-0.34, -0.04]	0.016
Loewenthal (2021)	-0.18 [-0.33, -0.02]	0.024
Pandit (2022)	-0.17 [-0.32, -0.01]	0.032
Peterson (2021)	-0.19 [-0.34, -0.04]	0.014
Purdie (2023)	-0.18 [-0.33, -0.02]	0.023
Schmeusser (2023)	-0.16 [-0.31, -0.01]	0.035
Verweij (2017)	-0.19 [-0.35, -0.04]	0.015
Weitzman (2021)	-0.17 [-0.32, -0.02]	0.031
Seeland (2024)	-0.18 [-0.33, -0.02]	0.025

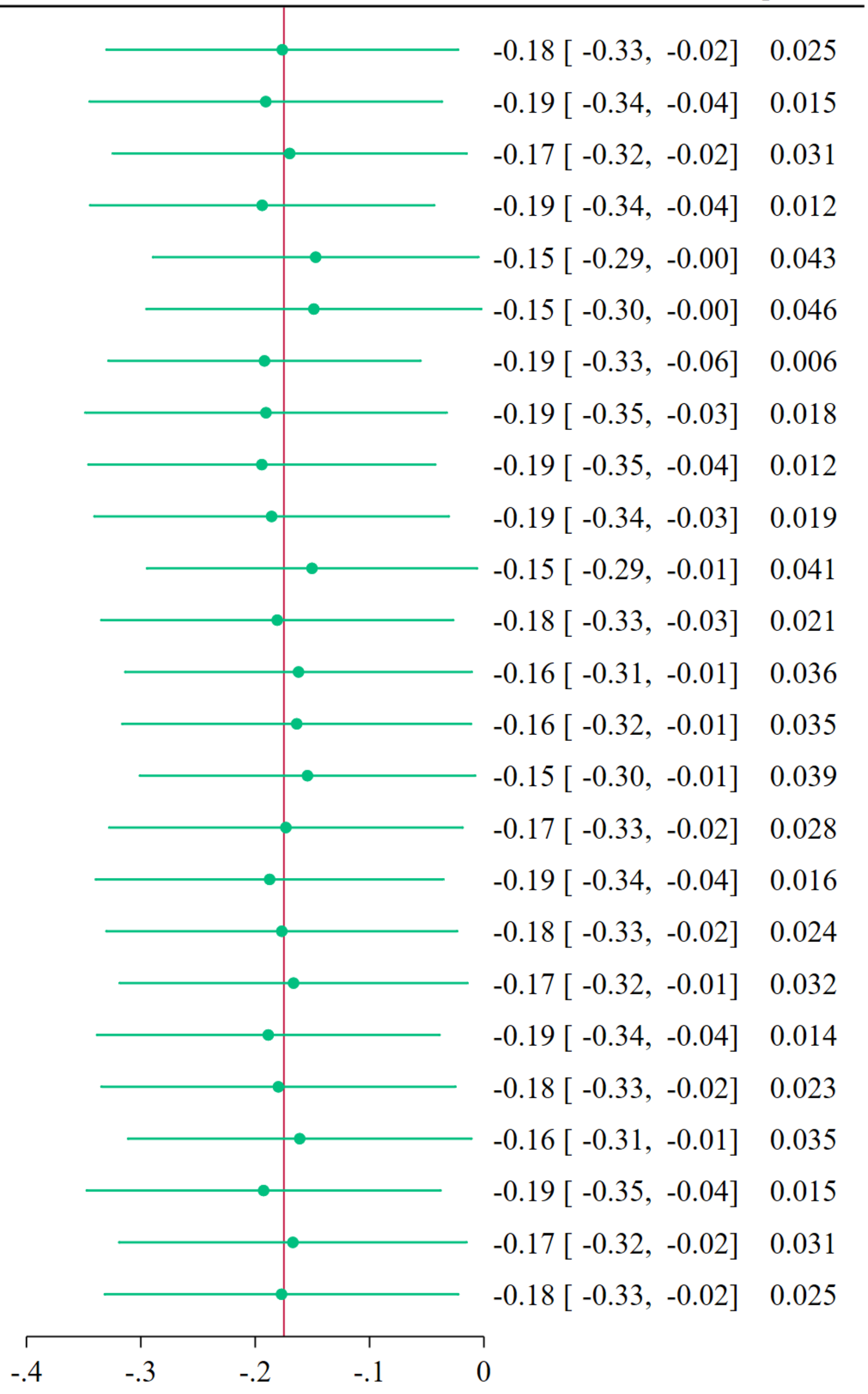

Random-effects DerSimonian–Laird model

Supplementary Appendix 3.3. Sensitivity analyses by leave-one-out method for standardized mean difference of PA score in individual intervention studies

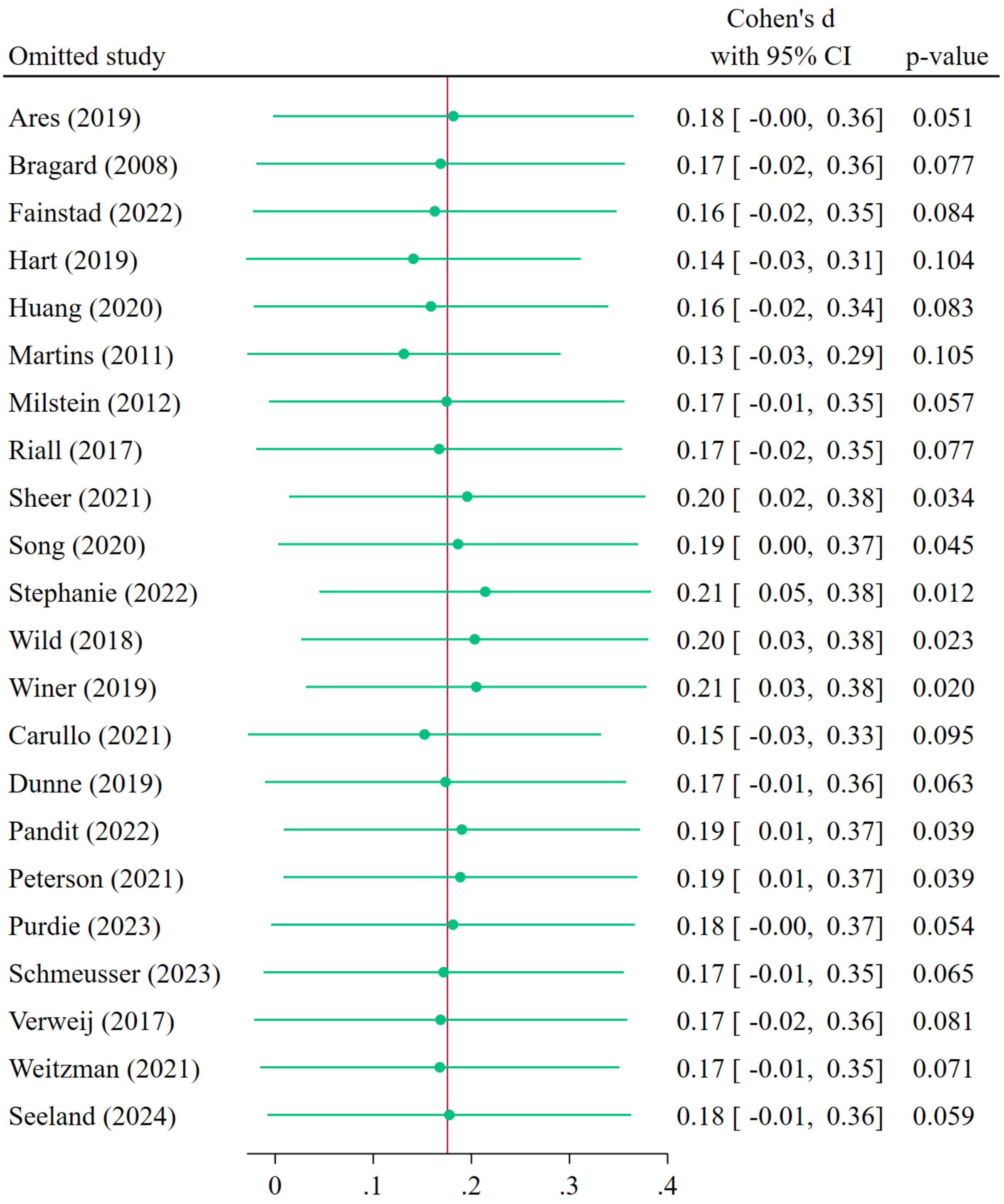

Random-effects DerSimonian–Laird model

Supplementary Appendix 3.4. Sensitivity analyses by leave-one-out method for standardized mean difference of EE score in organizational intervention studies

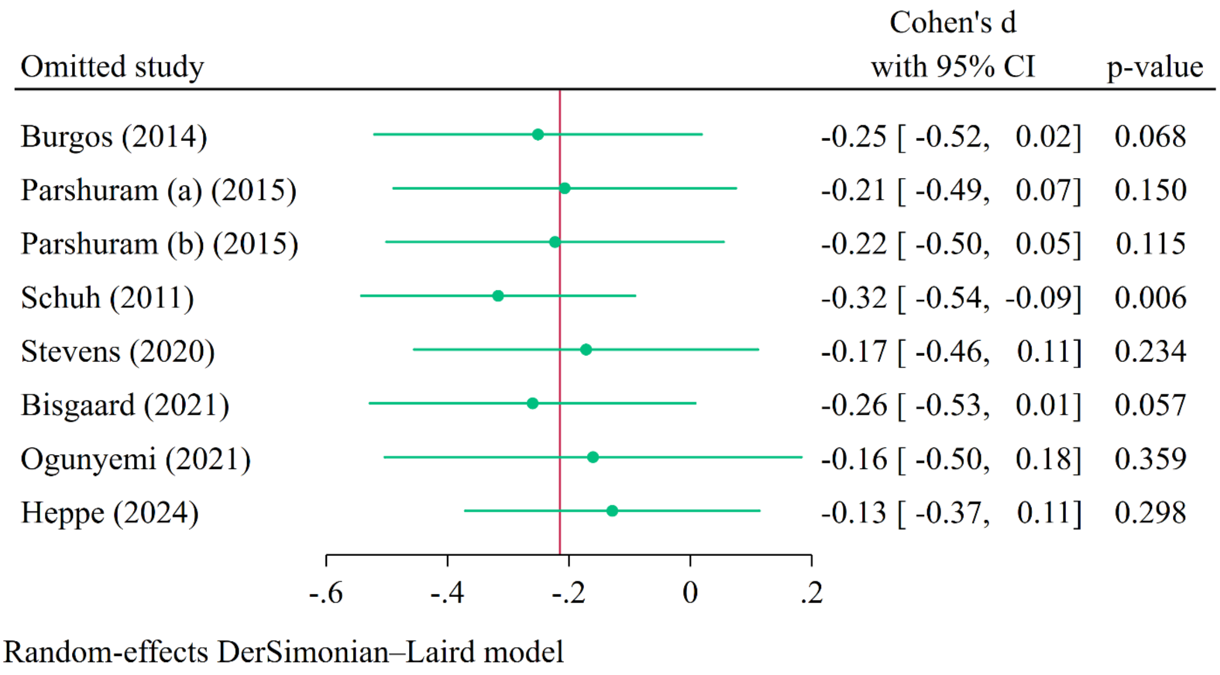

Supplementary Appendix 3.5. Sensitivity analyses by leave-one-out method for standardized mean difference of DP score in organizational intervention studies

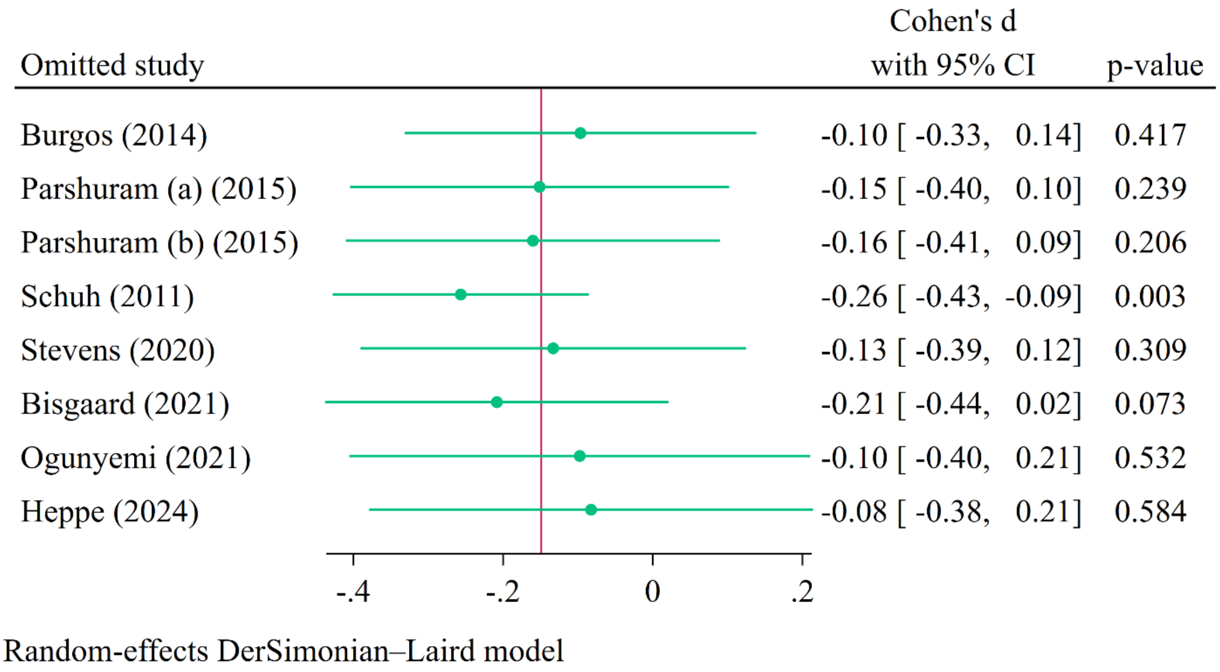

Supplementary Appendix 3.6. Sensitivity analyses by leave-one-out method for standardized mean difference of PA score in organizational intervention studies

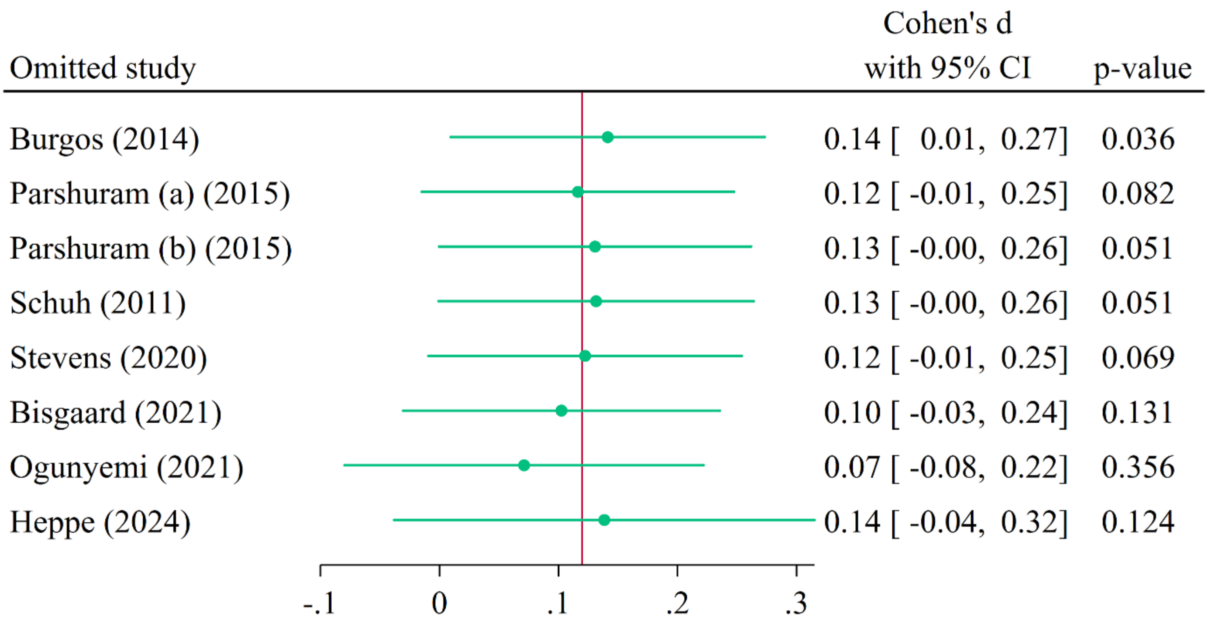

Random-effects DerSimonian–Laird model

Supplementary Appendix 4.1. Funnel plot of standardized mean difference of EE score in individual intervention studies

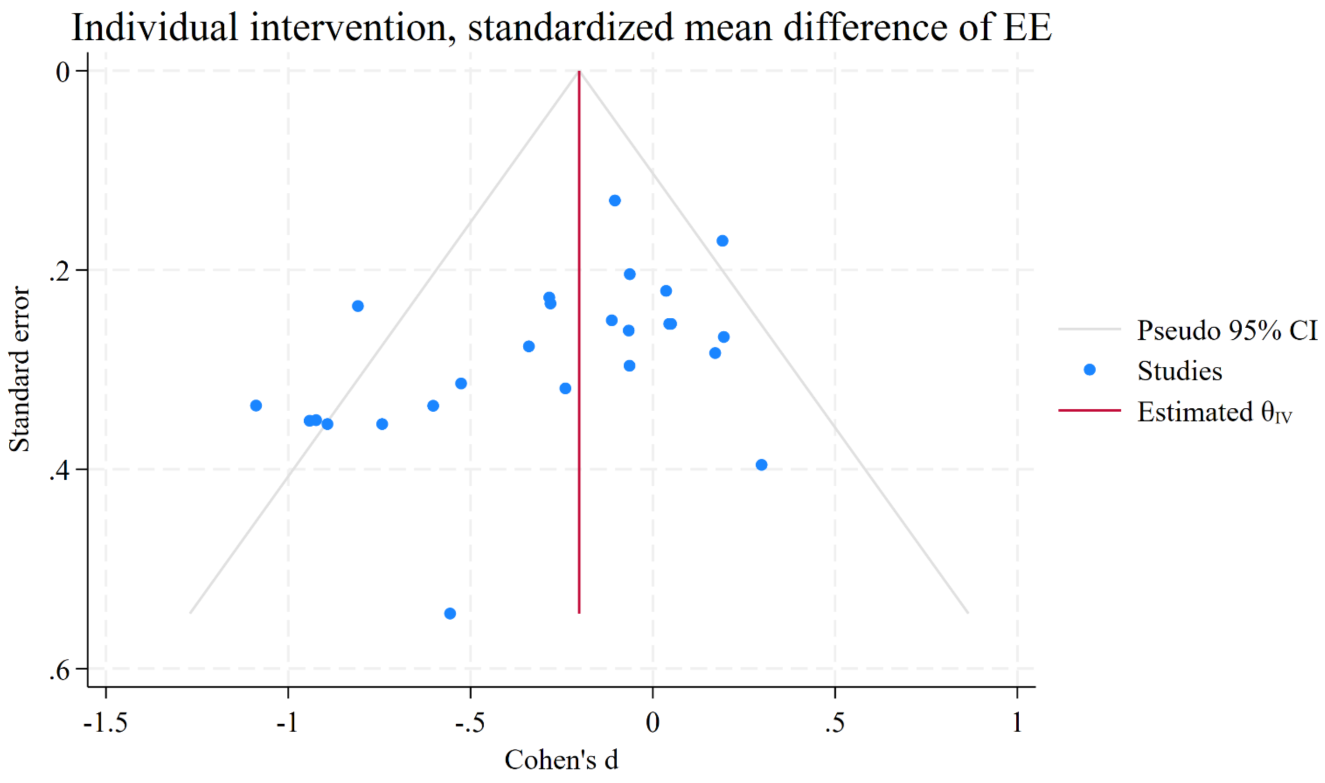

Supplementary Appendix 4.2. Funnel plot of standardized mean difference of DP score in individual intervention studies

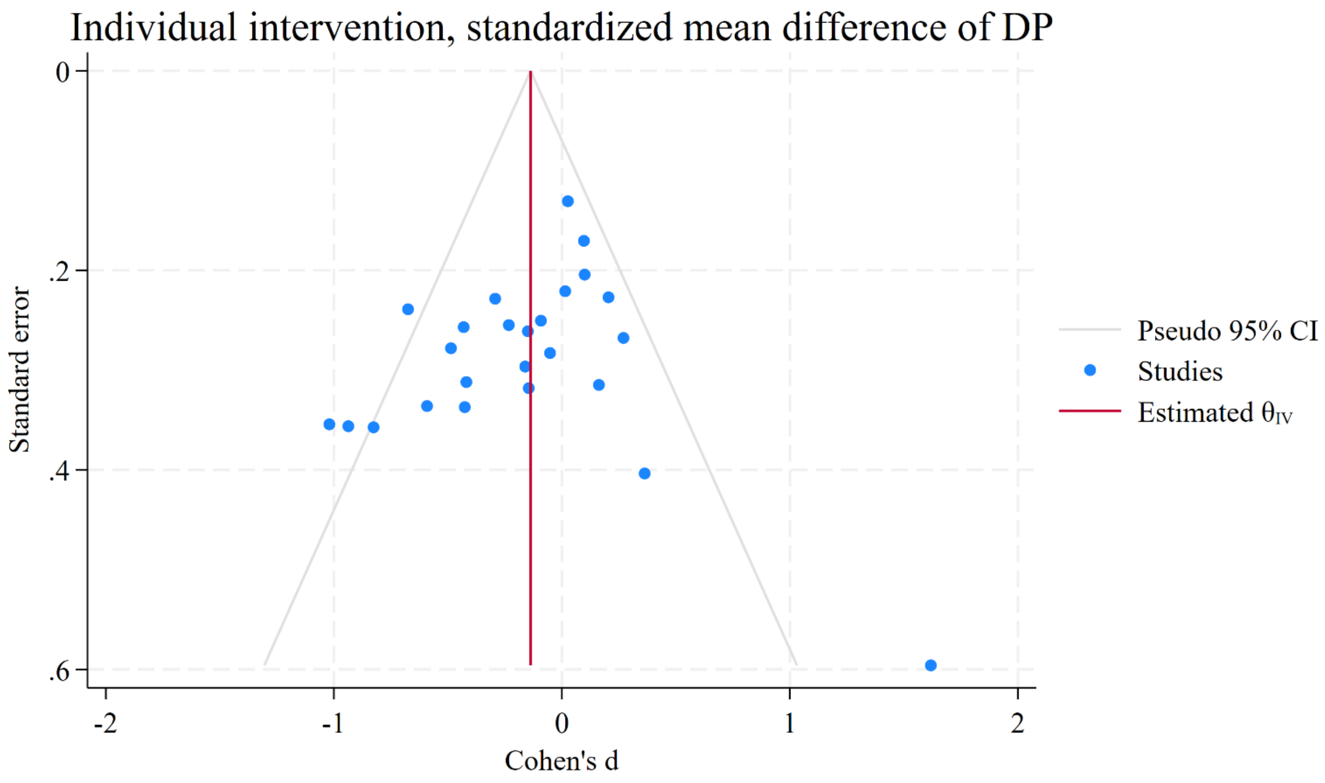

Supplementary Appendix 4.3. Funnel plot of standardized mean difference of PA score in individual intervention studies

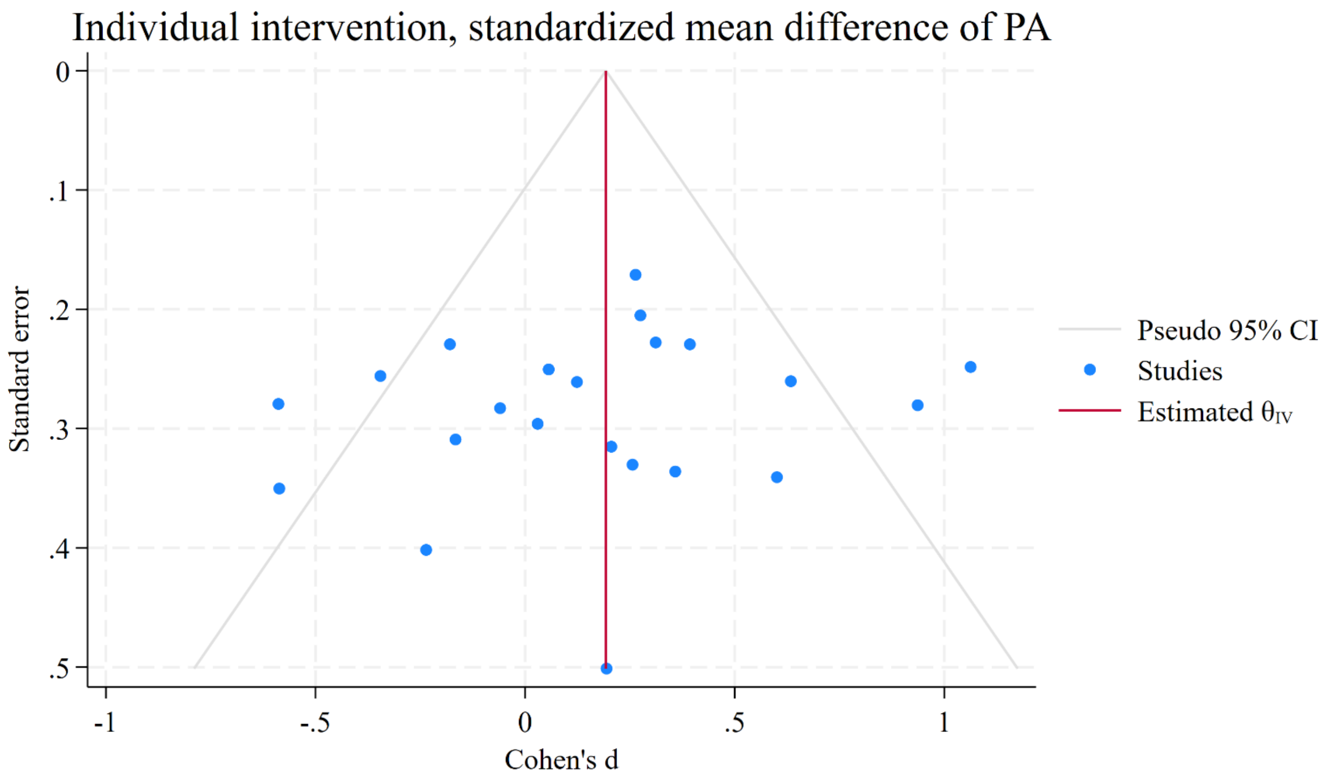

Supplementary Appendix 4.4. Funnel plot of standardized mean difference of EE score in organizational intervention studies

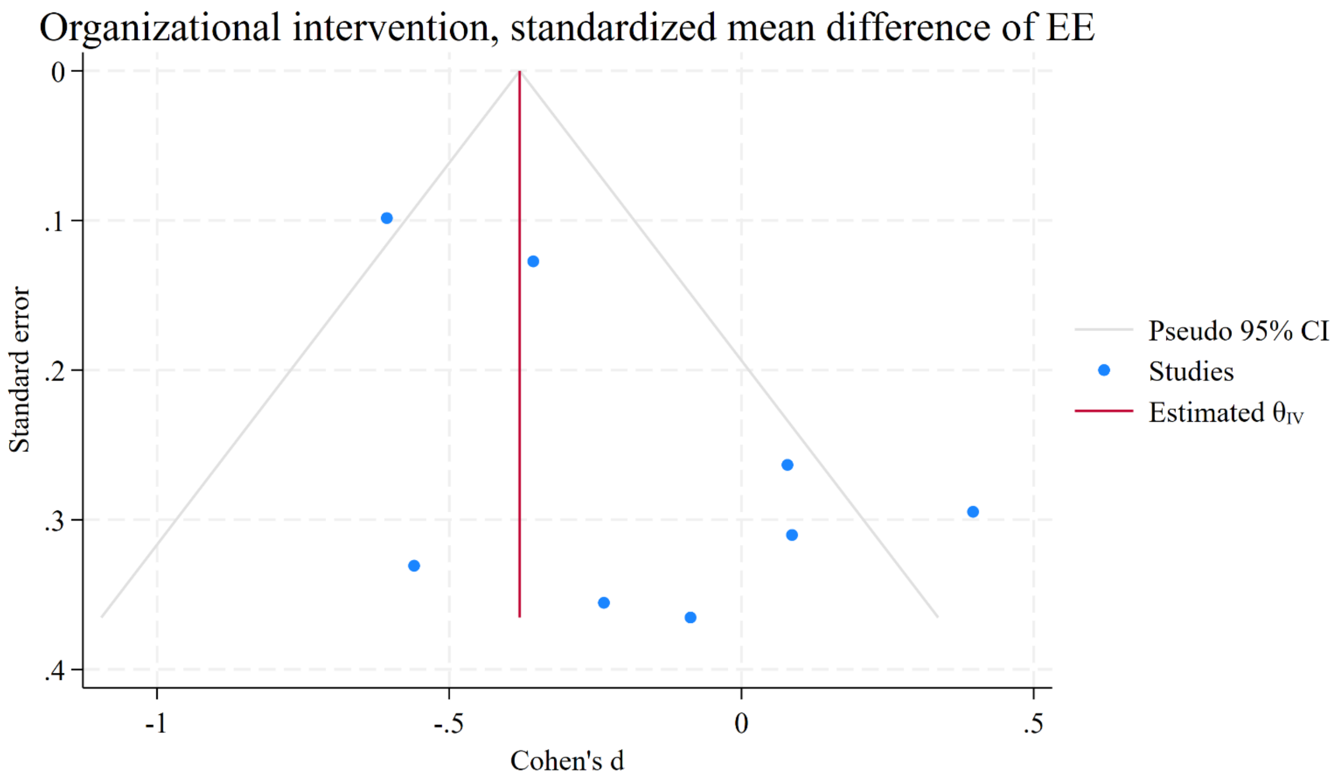

Supplementary Appendix 4.5. Funnel plot of standardized mean difference of DP score in organizational intervention studies

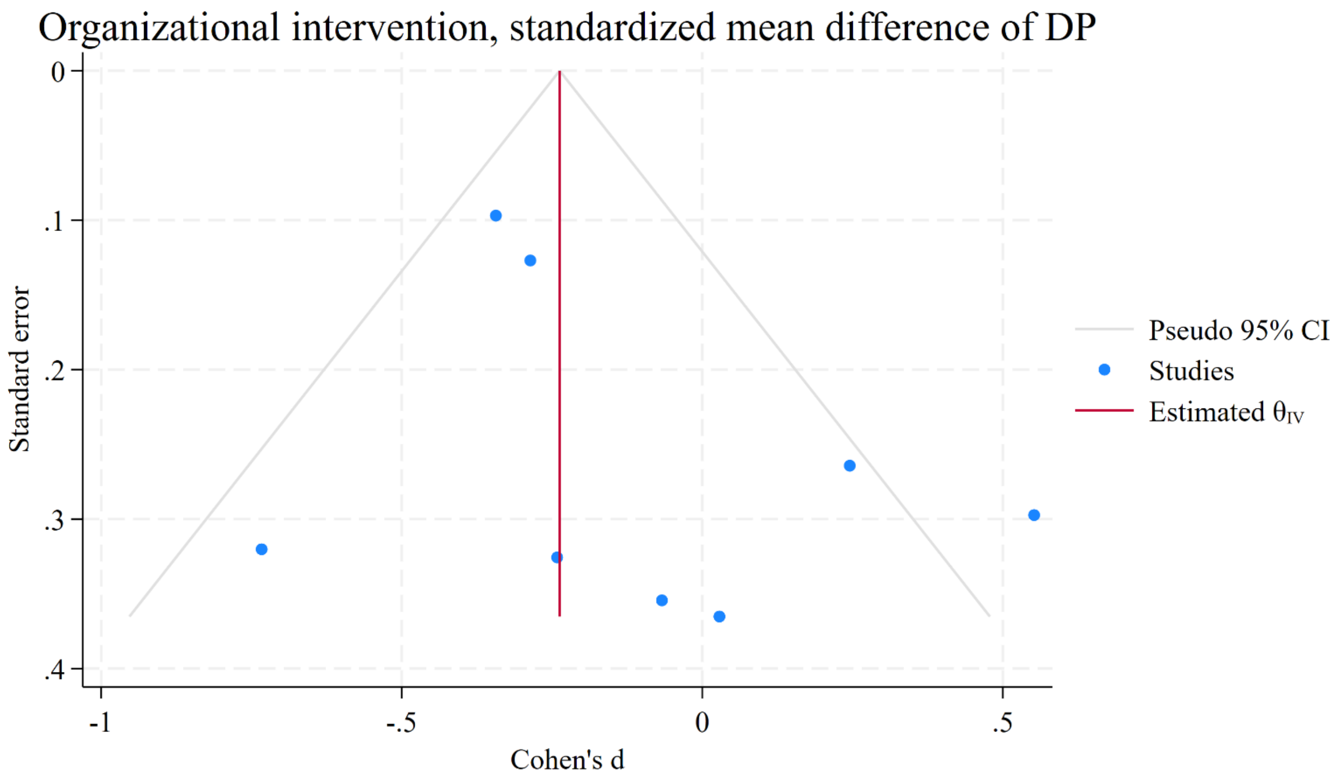

Supplementary Appendix 4.6. Funnel plot of standardized mean difference of PA score in organizational intervention studies

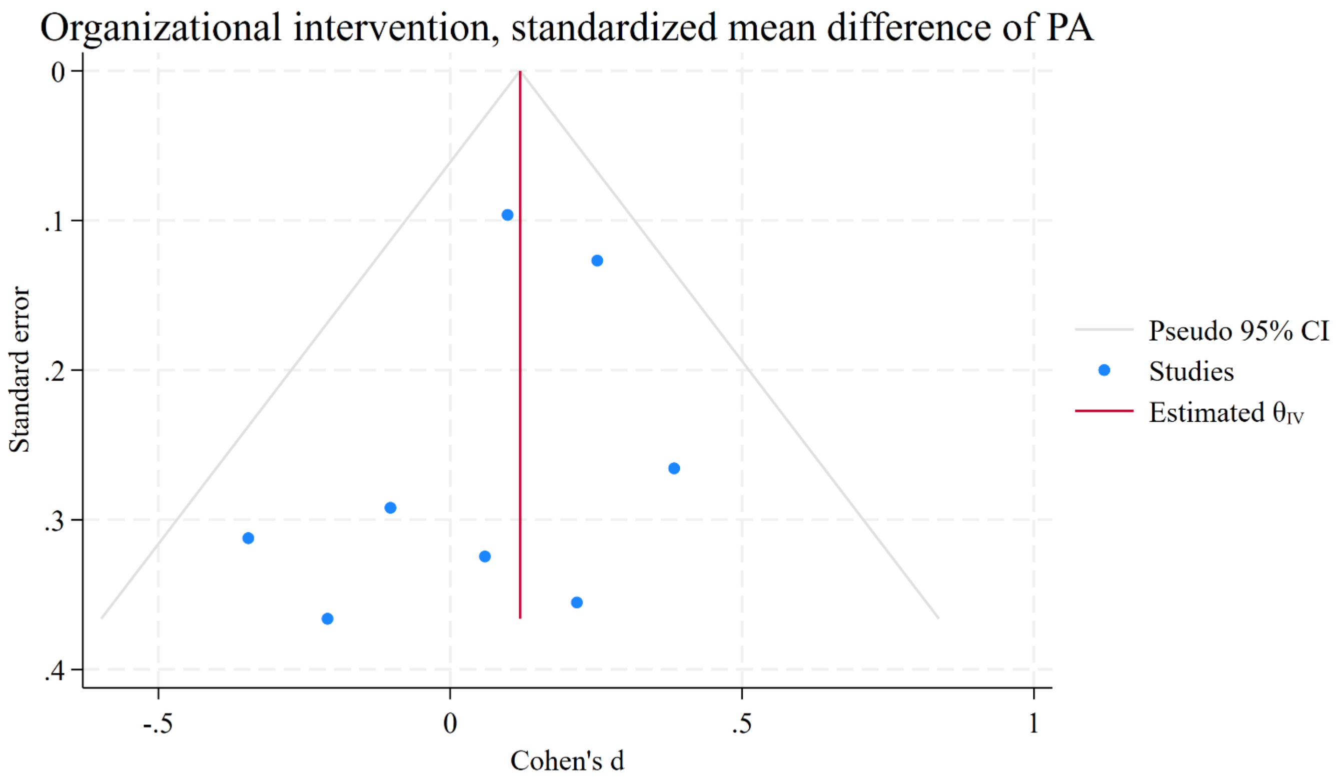

Supplementary Appendix 5.1. Summary of the risk of bias assessment by Cochrane's RoB2 across 11 randomized controlled concurrent studies

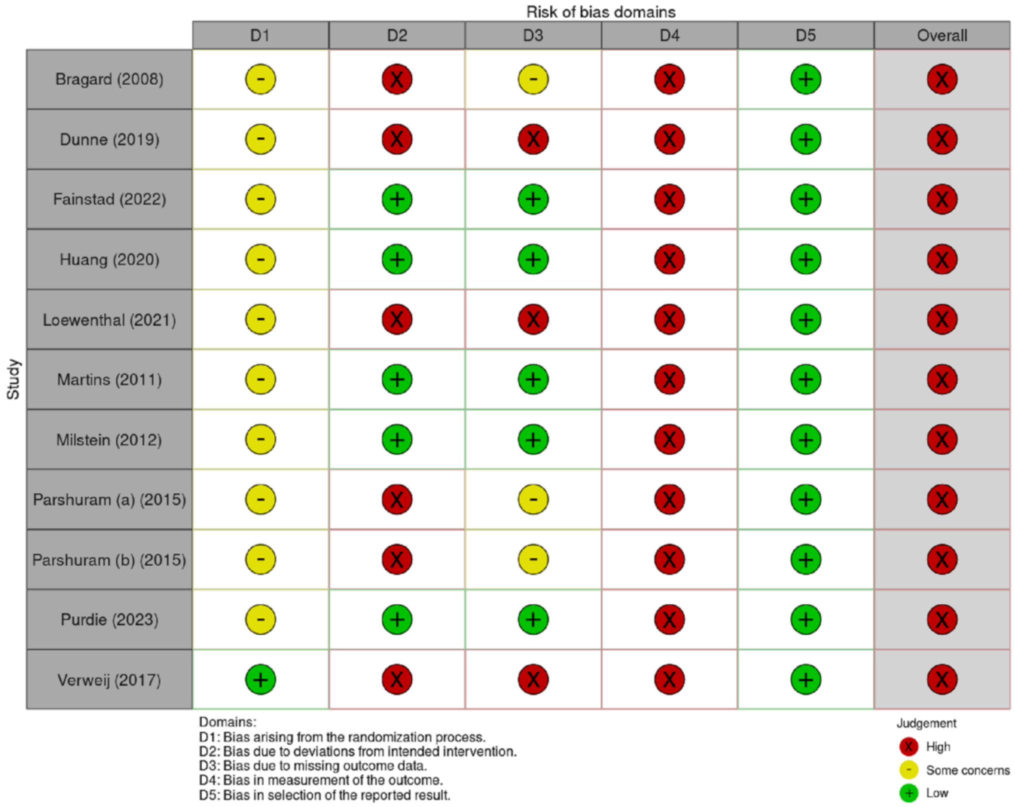

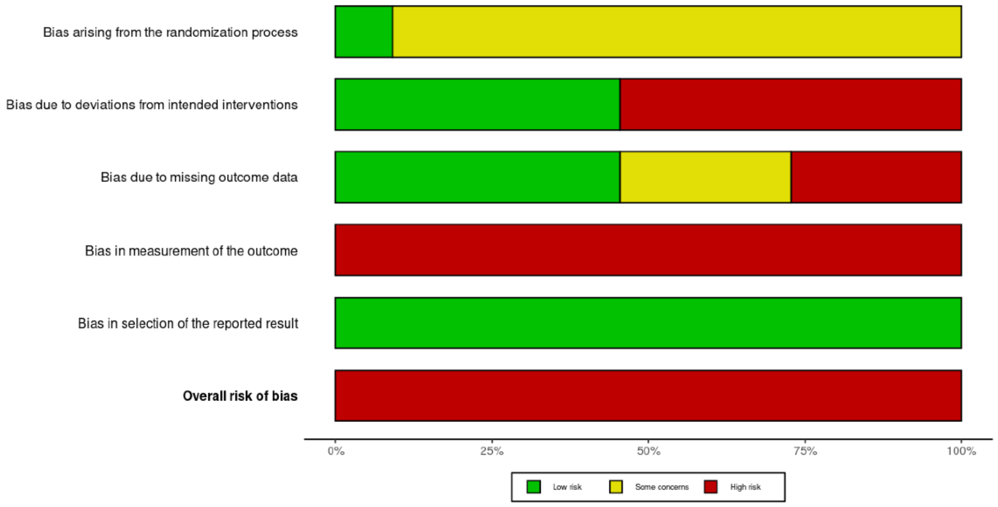

Supplementary Appendix 5.2. Summary of the risk of bias assessment by Cochrane's ROBINS-I across 22 non-randomized studies

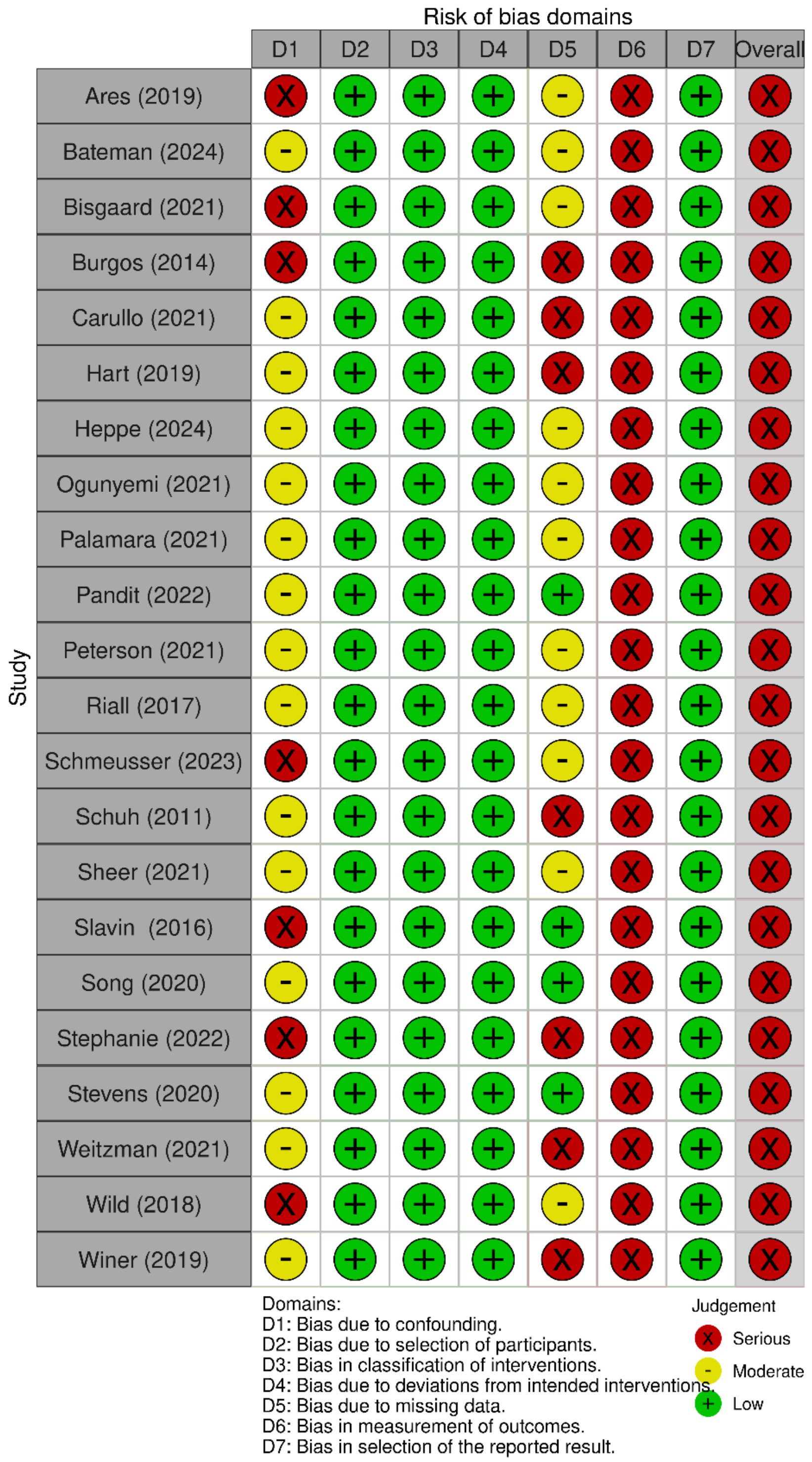

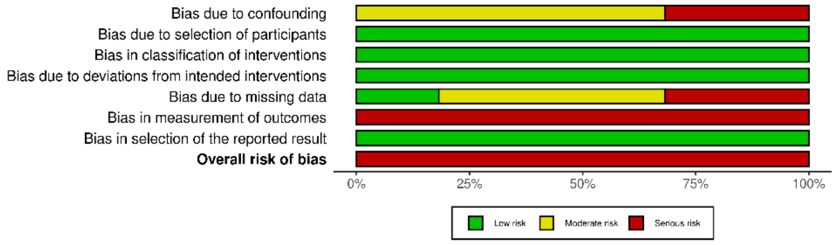

Supplement: Supplementary file 1 — Supplementary Material 1 [file 12909_2024_6195_MOESM1_ESM.pdf]
